# On an objective, geometrically exact coupling element for a director-based multi-body finite element framework

**DOI:** 10.1007/s11044-024-09998-w

**Published:** 2024-06-05

**Authors:** David Märtins, Daniel Schuster, Christian Hente, Cristian Guillermo Gebhardt, Raimund Rolfes

**Affiliations:** 1 Institute of Structural Analysis, Leibniz Universität Hannover, ForWind, Appelstr. 9A, Hannover, 30167 Germany; 2 Bergen Offshore Wind Centre (BOW), University of Bergen, Allégaten 70, Bergen, 5007 Norway

**Keywords:** Director-based kinematics, Node-to-node coupling element, Objectivity, Geometrically exact deformation

## Abstract

In multi-body systems, flexible components and couplings between them can be subject to large displacements and rotations. This contribution presents a general objective and geometrically exact node-to-node coupling element that pursues two innovations. Firstly, the coupling element represents a consistent extension to an existing nonlinear mechanical framework. The coupling element is intended to preserve its attributes of objectivity, path independence and adherence to the energy-conserving or energy-dissipative time integration method. Secondly, beside elasticity, inertia and damping properties are also considered. For this purpose, a director-based formulation is employed within a total Lagrangian description. The avoidance of an angle-based representation, along with the additive updating of state variables, results not only in path independence but also in the avoidance of cumulative errors during extended simulations. An objective deformation measure is chosen based on the Green–Lagrange strain tensor. The inertia forces are considered by an arbitrarily shaped continuum located at the centre of the coupled nodes. Damping is considered by using two different objective first-order dissipation functions, which further ensure energy conservation or dissipation. We successfully demonstrate the coupling element within the mechanical framework on using example applications. Firstly, the geometrically exact behaviour is shown compared to a linear deformation measure. Secondly, we numerically show the path independence of the formulation. The dynamic behaviour is demonstrated in a transient analysis of a damped structure. Finally, the modal analysis of a wind turbine shows the application of the coupling element to model the soil–structure interaction.

## Introduction

Flexible couplings between components in multi-body systems (MBSs) and structural systems can exhibit complex mechanical behaviour. The investigation of detailed properties is a challenging task, necessitating sophisticated models or experiments. To achieve a balance between computational efficiency and accurate global behaviour approximation, the properties derived from detailed investigations can be incorporated into a coupling element. This can then be used to model MBS and structural systems. Especially when examining the nonlinear behaviour of mechanical models, it is crucial to also consider the geometrically nonlinear characteristics of its flexible couplings.

The motivation of this work is twofold. First, there is a need for a mechanical framework that can represent the nonlinear behaviour of structures. Second, the development of a general coupling element that equally models the geometric nonlinearity of connections without violating the consistency of the mechanical framework is necessary.

Geometric nonlinearities of complex mechanical systems consisting of slender structural members can be modelled with appropriate nonlinear MBS. Such structures include for example slender and elastic aircraft wings, in which the occurring geometrical nonlinearity significantly affects the dynamic behaviour of the wing and thus the aircraft itself [1]. Another example is provided by energy converter turbines, in which turbine blades are being developed to be ever more slender due to increasing rotor sizes. Here, nonlinear geometric behaviour also has to be taken into account [2].

Moreover, couplings in these MBS can undergo large displacements and rotations. To model their mechanical behaviour, geometrically nonlinearities again have to be considered. An example of couplings undergoing nonlinear geometrical deformation can be found in the adhesive lap joints discussed by Andruet et al. [3]. These are subject to important geometric nonlinear effects due to eccentrically applied forces. Dispersyn et al. [4] investigate adhesive point fixings in structural glass facades, considering nonlinear geometric deformations and material nonlinearities. Similarly, in [5], a study of adhesively bonded composites and their thermal effects reveals that small strains and large displacements accurately represent the joint behaviour. Geometric nonlinearities also arise in the deformation of rubber bushings in automotive suspensions, as outlined in [6]. In MBS frameworks that analyse structural behaviour, these examples can be simplified into a coupling element with the appropriate properties.

In recent decades, MBS approaches were developed to consider nonlinear mechanical behaviour. The commonly used wind-turbine-specific codes [7–9] use angle-based geometrically exact beams to model slender components. This formulation, in combination with an updated Lagrangian description, does not maintain objectivity and path independence. Borri et al. [10] present an invariant-preserving approach that maintains objectivity. This consists of rigid bodies, geometrically exact beams, shell elements and kinematic joints, and is formulated in an angle-based way. Betsch et al. [11] and Romero et al. [12] introduce director-based geometrically exact beam formulations in a total Lagrangian description. Their formulations are objective and preserve the total energy of the system. Furthermore, the director-based formulation, in combination with additive updating of the rotation, leads to path independence. Gebhardt et al. developed an MBS formulation, consisting of the three canonical models rigid bodies, geometrically exact beams, and solid-degenerate shells [13–18]. Also, director-based kinematics in a total Lagrangian formulation are used to maintain objectivity and path independence. The total energy of a mechanical system is preserved or dissipated using a time integration scheme based on the midpoint rule and the *average-vector-field* method [19]. Singularities that might occur in the stiffness matrices formulated in [11] and [12] are avoided, allowing buckling analyses [20]. The presented coupling element is developed as a consistent contribution to this mechanical MBS framework.

Coupling elements are implemented in commonly used MBSs and finite element (FE) codes. For instance, the well-established FE code ABAQUS [21] allows the use of linear translational and rotational spring elements (*SPRING, *JOINTC) to model node-to-node connections or to couple one node with the surrounding environment. Similarly, the multi-body dynamics software MSC ADAMS [22] provides integration options for *BUSHING* and *FIELD* elements, which calculate forces and moments acting on connected nodes based on a linear deformation measure. These coupling elements assume small deformations.

To address the limitations of linear deformation measures and enable the modelling of large displacements and rotations, Masarati and Morandini [23] developed a formulation that represents constitutive relations for lumped deformable components experiencing finite deformations. They found that nonlinear constitutive laws must be considered when strains are not small. However, large relative displacements and geometric nonlinear behaviour do not necessarily imply large strains. Bauchau [24] introduces a family of finite deformation measures suitable for characterising moderate deformations of flexible joints using angle-based kinematics. He demonstrates that combining tensorial deformation measures with linear constitutive laws accurately predict moderate deformations. An extensive derivation of an objective angle-based formulation is presented.

The newly developed coupling element described in this paper incorporates the following two main innovations. First, the coupling element is a consistent extension of the presented mechanical MBS and FE framework. It is objective under rigid body motion, maintains path independence and preserves linear and angular momentum as well as the total energy of the overall system. Fully nonlinear static and dynamic analyses can be carried out. Second, the element considers mass and damping without compromising the desired properties. To the best of our knowledge, there is no literature on a general coupling element that considers elasticity, mass and damping in a single element. Masses that cause inertia forces are modelled as an arbitrary continuum between the nodes concerned. The objective strain/stress-dependent damping formulation derived by Armero and Romero [25] and Gebhardt et al. [19] is introduced. Additionally, we demonstrate the possibility of using dissipation functions tailored to specific physical problems, such as obtaining a strain-rate/stress-rate dependent damping.

The work is structured as follows: in Sect. 2 the main ideas behind the mechanical framework are presented. The equations incorporating the stiffness, mass and damping properties behind the coupling element are derived in Sect. 3. In Sect. 4, application examples are given to illustrate the behaviour of the coupling element. Finally, a summary of the work together with limiting aspects and an outlook on future work is given in Sect. 5.

## Mechanical framework

The general coupling element presented in this work is a consistent extension of the mechanical MBS framework developed by Gebhardt et al. [13–20]. The framework uses a mixed displacement-velocity-strain-based formulation and director-based kinematics in a total Lagrangian description. It comprises three canonical models: rigid bodies, geometrically exact beams and solid-degenerate shells. Beams and shells are discretised using the FE method. The subsequent section describes the fundamental equations and main ideas of the framework and briefly shows the spatial and temporal discretisations. For more details, we refer the reader to the aforementioned literature.

### Continuous governing equation

Based on the primal-dual variational principle, for a material body $\mathcal{B}_{0} \subseteq \mathbb{R}^{3}$, the continuous governing equation of the constrained system is determined by

1 $$\begin{aligned} \begin{aligned} \delta S = \int _{\mathcal{B}_{0}} & \bigl(\langle \delta \mathbf{v}, \mathbf{l}(\mathbf{v},t)- \mathbf{l}(\dot{\mathbf{x}},t) \rangle + \langle \delta \boldsymbol{\lambda}, \mathbf{h}(\mathbf{x},t)\rangle \\ & + \langle \delta \mathbf{x}, \dot{\mathbf{l}}(\mathbf{v},t) + \mathbf{f}^{\text{int}}(\mathbf{x},t) - \mathbf{f}^{\text{ext}}(t) + \mathbf{H}(\mathbf{x},t)^{\text{T}}\boldsymbol{\lambda}(t) \rangle \bigr) dV=0. \end{aligned} \end{aligned}$$

Herein $\langle \cdot ,\cdot \rangle $ denotes a scalar product and $\delta (\cdot )$ the admissible variation of a given quantity. The positions and velocities of a material point are denoted by $\mathbf{x}(\theta _{i}, t) = \boldsymbol{\Theta }^{\text{T}} \mathbf{q}$ and $\mathbf{v}(\theta _{i}, t) = \boldsymbol{\Theta }^{\text{T}} \mathbf{s}$, respectively. These specifically depend on the canonical model. $\theta _{i}$, with $i= 1,2,3$, specifies the coordinates of a material point in the director coordinate system of a reference point of a canonical model. $\boldsymbol{\Theta }^{\text{T}} = [\mathbf{I}, \theta _{1}\mathbf{I}, \theta _{2}\mathbf{I},\theta _{3}\mathbf{I}] \in \mathbb{R}^{3\times 12}$ contains the director coordinates. $\mathbf{q}$ and $\mathbf{s}$ are vectors of the generalised coordinates and velocities, considering the kinematics. As a mixed formulation is used, the equivalence of the displacement-based and velocity-based momentum densities is incorporated into Equation (1) by using $\mathbf{l}(\dot{\mathbf{x}},t)$ and $\mathbf{l}(\mathbf{v},t)$, respectively. Inertia terms are taken into account by using the time derivative of the velocity-based momentum density $\dot{\mathbf{l}}(\mathbf{v},t)$. $\mathbf{f}^{\text{int}}(\mathbf{x},t)$ and $\mathbf{f}^{\text{ext}}(t)$ are the internal and external force densities, respectively. $\mathbf{h}(\mathbf{x},t)$ represents a set of kinematic restrictions and $\boldsymbol{\lambda}(t)$ is a vector of Lagrange-multipliers. $\mathbf{H}(\mathbf{x},t)$ is the Jacobian of the holonomic constraints.

A three-director formulation is chosen, with the directors denoted by $\mathbf{d}_{i}$ and $i = 1,2,3$. The additive update of the rotation in the total Lagrangian description ensures the path independence. With the outer product ⋅⊗⋅ and the standard Euclidean basis $\mathbf{e}_{i}$, the rotational tensor $\mathbf{R}$ is represented by

2 $$\begin{aligned} \mathbf{R} = \mathbf{d}_{1} \otimes \mathbf{e}_{1} + \mathbf{d}_{2} \otimes \mathbf{e}_{2} + \mathbf{d}_{3} \otimes \mathbf{e}_{3}. \end{aligned}$$

As we will refer to the kinetic and elastic strain energy later, they are subsequently introduced. The mass density per unit volume $\rho $ and the constant symmetric mass matrix $\mathbf{M}$ lead to the kinetic energy $T$:

3 $$\begin{aligned} T = \frac{1}{2}\int _{\mathcal{B}_{0}}\rho \langle \mathbf{v}(\theta _{i},t), \mathbf{v}(\theta _{i},t)\rangle dV. \end{aligned}$$

The virtual work $\delta W$ of the mechanical system is given by

4 $$\begin{aligned} \delta W =\int _{\mathcal{B}0} \langle \delta \mathbf{x}, \mathbf{f}^{ \text{int}}(\mathbf{x},t)\rangle dV = \int _{\mathcal{B}_{0}} \langle \delta \mathbf{E}(\mathbf{x}), \mathbf{S}\rangle dV . \end{aligned}$$

Herein $\mathbf{E}$ denotes the Green–Lagrange strain tensor and $\mathbf{S}$ the corresponding work-conjugated stress tensor, here the second Piola–Kirchhoff stress tensor. We refer the reader to [26]:

5 $$\begin{aligned} \mathbf{E} &= \frac{1}{2}(\mathbf{F}(t)^{\text{T}}\mathbf{F}(t)- \mathbf{F}(0)^{\text{T}}\mathbf{F}(0)), \end{aligned}$$

with $\mathbf{F}$ being the tangent map (also referred to as deformation gradient). Of course, no elastic energy is stored in rigid bodies. For a more detailed discussion of the geometrically exact beam analysis, we refer to [27] and [28].

### Discretisation of the governing equation in space and time

To spatially and temporally discretise Equation (1), we first have to substitute the coordinates and velocities $\mathbf{x}$ and $\mathbf{v}$ by using a set of generalised coordinates and velocities $\mathbf{q}$ and $\mathbf{s}$. Here, the kinematics of the respective canonical model are taken into account, as explained in [28].

Equation (1) is spatially discretised using the FE method. We use two-node elements and linear Lagrange-type shape functions. The solid-degenerate shell elements use bi-linear shape functions and two-point Gaussian quadrature in the thickness direction. To maintain the orthogonality and the initial length $||\mathbf{d}_{i}(0)||=1$ of the director triad in each node, a set of internal constraints is applied, as used in [14].

The framework employs an implicit time integration scheme. It is based on the midpoint rule and the *average-vector-field* method and preserves the linear and angular momentum as well as the total energy of the system to ensure physical correctness and robustness. For a detailed description of the temporal discretisation, see [14], where this method is discussed extensively.

Following the midpoint rule, the configuration and generalised velocities are approximated in between two time steps $t_{n+\frac{1}{2}} = \frac{1}{2}(t_{n+1}-t_{n})$, using the time steps $n$ and $n+1$ and the time step size $\Delta t = t_{n+1}-t_{n}$, as follows:

6 $$\begin{aligned} \mathbf{q}_{t_{n+\frac{1}{2}}} \approx \frac{\mathbf{q}_{t_{n}}+\mathbf{q}_{t_{n+1}}}{2}, \quad \mathbf{s}_{t_{n+ \frac{1}{2}}} \approx \frac{\mathbf{s}_{t_{n}}+\mathbf{s}_{t_{n+1}}}{2}. \end{aligned}$$

The evaluation of the governing equation, using Equation (6), leads to its spatially and temporally discrete form, presented as Equation (7). The superscript $(\cdot )^{d}$ indicates spatially discrete quantities that were introduced in the continuous context in Equation (1).

7 $$ \begin{aligned} \delta S_{t_{n+1}}^{d} = &\bigl(\langle \delta \mathbf{s}^{d}_{t_{n+1}}, \mathbf{l}^{d}(\mathbf{s}^{d}_{t_{n+1}}, \mathbf{s}^{d}_{t_{n}})-\mathbf{l}^{d}(\dot{\mathbf{q}}^{d}_{t_{n+1}}, \dot{\mathbf{q}}^{d}_{t_{n}}) \rangle + \langle \delta \boldsymbol{\lambda}^{d}_{t_{n+1}},\mathbf{h}^{d}(\mathbf{q}_{t_{n+1}}, \mathbf{q}^{d}_{t_{n}})\rangle \\ &+ \langle \delta \mathbf{q}^{d}_{t_{n+\frac{1}{2}}}, \dot{\mathbf{l}}^{d}(\mathbf{s}^{d}_{t_{n+1}}, \mathbf{s}^{d}_{t_{n}}) + \mathbf{f}^{\text{int,d}}(\mathbf{q}^{d}_{t_{n+1}},\mathbf{q}^{d}_{t_{n}}) - \mathbf{f}^{\text{ext,d}}(\mathbf{q}^{d}_{t_{n+1}},\mathbf{q}^{d}_{t_{n}}) \\ &+ {\mathbf{H}^{d}}^{\text{T}}(\mathbf{q}^{d}_{t_{n+1}}, \mathbf{q}^{d}_{t_{n}}) \boldsymbol{\lambda}^{d}_{t_{n+\frac{1}{2}}} \rangle \bigr) =0, \end{aligned} $$

see also [14]. In the discretised form, we consider nonconservative forces in the discrete vector of internal forces $\mathbf{f}^{\text{int},d}(\mathbf{q}^{d}_{t_{n+1}}, \mathbf{q}^{d}_{t_{n}})$ as strain/stress dependent energy dissipation can be added to the geometrically exact beams and the solid-degenerate shells. Additionally, velocity-based dissipation can be applied to the beam and shell elements. Accordingly, the velocity-based momentum $\mathbf{l}^{d}(\mathbf{s}^{d}_{t_{n+1}},\mathbf{s}^{d}_{t_{n}})$ considers nonconservative components also. We refer the reader to the derivations in [19, 25]. Equation (7), in its discrete form, can also contain nonconservative forces in $\mathbf{f}^{\text{ext},d}(\mathbf{q}^{d}_{t_{n+1}},\mathbf{q}^{d}_{t_{n}})$. Although it is theoretically possible to consider higher-order dissipation functions, we restrict ourselves to first-order dissipation functions. As we exclusively consider spatially and temporally discrete quantities from now on, the superscript $(\cdot )^{d}$ is omitted again. Equation (7) has to be true for arbitrary variations, which leads to the nonlinear spatially and temporally discrete governing equation of the mechanical framework

8 $$\begin{aligned} \mathbf{g}(\mathbf{q}_{t_{n+1}},\mathbf{s}_{t_{n+1}}, \boldsymbol{\lambda}_{t_{n+1}}) = \left [ { \textstyle\begin{array}{c} \mathbf{f}^{\text{int}}(\mathbf{q})- \mathbf{f}^{\text{ext}}(\mathbf{q}) +\dot{\mathbf{l}}(\mathbf{s})+\mathbf{H}^{\text{T}}(\mathbf{q}) \boldsymbol{\lambda} \\ \mathbf{l}(\mathbf{s})-\mathbf{l}(\mathbf{q}) \\ \mathbf{h}(\mathbf{q}) \end{array}\displaystyle } \right ]_{t_{n+1}} = \mathbf{0}. \end{aligned}$$

### Solution algorithm

The unknown state variables in the nonlinear governing Equation (8) are the generalised coordinates $\mathbf{q}_{t_{n+1}}$, the generalised velocities $\mathbf{s}_{t_{n+1}}$ and the Lagrange-multipliers $\boldsymbol{\lambda}_{t_{n+1}}$, each at the next time step $t_{n+1}$. This nonlinear governing equation is solved iteratively using Newton’s method. We solve for the unknown relative increments $\Delta \mathbf{q}$, $\Delta \mathbf{s}$ and $\Delta \boldsymbol{\lambda}$ at the iteration step $k$

9 $$\begin{aligned} \mathbf{g}(\mathbf{q}_{t_{n+1}},\mathbf{s}_{t_{n+1}}, \boldsymbol{\lambda}_{t_{n+1}})^{k+1} = & \mathbf{g}(\mathbf{q}_{t_{n+1}}, \mathbf{s}_{t_{n+1}},\boldsymbol{\lambda}_{t_{n+1}})^{k} \\ &+\Delta _{G} \mathbf{g}(\mathbf{q}_{t_{n+1}},\mathbf{s}_{t_{n+1}}, \boldsymbol{\lambda}_{t_{n+1}})^{k} = \mathbf{0}. \end{aligned}$$

Here, $\Delta _{G}(\cdot )=\frac{\partial (\cdot )}{\partial \mathbf{q}} \Delta _{k} \mathbf{q} +\frac{\partial (\cdot )}{\partial \mathbf{s}} \Delta _{k} \mathbf{s} + \frac{\partial (\cdot )}{\partial \boldsymbol{\lambda}}\Delta _{k} \boldsymbol{\lambda}$ is the Gâteaux derivative. The term $\Delta _{G}\mathbf{g}(\mathbf{q}_{t_{n+1}}, \mathbf{s}_{t_{n+1}}, \boldsymbol{\lambda}_{t_{n+1}})^{k}$ is consequently the incremental form of the nonlinear governing equation $\mathbf{g}(\mathbf{q}_{t_{n+1}},\mathbf{s}_{t_{n+1}}, \boldsymbol{\lambda}_{t_{n+1}})^{k}$ at the time instant $t_{n+1}$ and the iteration step $k$, following Newton’s method. Substituting the governing Equation (8) into the representation of Newton’s method (9), we obtain the following matrix formulation:

10 $$ \begin{aligned} \left [ { \textstyle\begin{array}{c} \mathbf{0} \\ \mathbf{0} \\ \mathbf{0} \end{array}\displaystyle } \right ] & = \left [ { \textstyle\begin{array}{c} \mathbf{f}^{\text{int}}(\mathbf{q})-\mathbf{f}^{\text{ext}}(\mathbf{q})+ \dot{\mathbf{l}}(\mathbf{s})+\mathbf{H}^{T}(\mathbf{q}) \boldsymbol{\lambda} \\ \mathbf{l}(\mathbf{s}) - \mathbf{l}(\mathbf{q}) \\ \mathbf{h}(\mathbf{q}) \end{array}\displaystyle } \right ]^{k}_{t_{n+1}} \\ & + \left [ { \textstyle\begin{array}{c@{\quad}c@{\quad}c} \mathbf{K}_{\text{int}}(\mathbf{q}) -\mathbf{K}_{\text{ext}}(\mathbf{q}) + \mathbf{K}_{\lambda \lambda}(\mathbf{q}) & \mathbf{K}_{qs} & \mathbf{H}^{\text{T}}(\mathbf{q}) \\ \mathbf{K}_{sq} & \mathbf{K}_{ss}(\mathbf{s}) & \mathbf{0} \\ \mathbf{H}(\mathbf{q}) & \mathbf{0} & \mathbf{0} \end{array}\displaystyle } \right ]^{k}_{t_{n+1}} \cdot \left [ { \textstyle\begin{array}{c} \Delta _{k}\mathbf{q} \\ \Delta _{k}\mathbf{s} \\ \Delta _{k}\boldsymbol{\lambda} \end{array}\displaystyle } \right ]. \end{aligned} $$

The tangent operator is composed as follows:

11 $$\begin{array}{rlrlrl} & K_{\text{int}}(q)=\frac{\partial f^{\text{int}}(q)}{\partial q}, & \; & K_{\text{ext}}(q)=\frac{\partial f^{\text{ext}}(q)}{\partial q}, & \; & K_{qs}=\frac{\partial \dot{l}(s)}{\partial s},\\ & K_{\lambda \lambda }(q)=\frac{\partial H^{\text{T}}(q)}{\partial q}, & & H^{\text{T}}(q)=\frac{\partial H^{\text{T}}(q)\lambda }{\partial \lambda }, & & K_{sq}=\frac{\partial l(q)}{\partial q},\\ & K_{ss}(s)=\frac{\partial l(s)}{\partial s}, & & H(q)=\frac{\partial h(q)}{\partial q}. \end{array}$$

$\Delta _{k} (\cdot ) =(\cdot )^{k+1}_{t_{n+1}} - (\cdot )^{k}_{t_{n+1}}$ denotes the change in a respective quantity between two iteration steps. If terms are added to the governing equation, this must of course be taken into account by adjusting its derivatives in the tangent operator to preserve the robust quadratic convergence rate of the solving algorithm [29].

## Derivation of the geometrically exact coupling element

In this section we derive the element equations according to Equations (8) and (9), i.e. all element forces and moments of the general node-to-node coupling element, including stiffness, mass and damping. The same element can also be utilised to couple a node to the surrounding environment. As stated previously, the mechanical equations for the coupling element provide a consistent extension of the mechanical framework described in [13–20] and briefly introduced in Sect. 2. This extension allows the geometrically exact consideration of flexible joints, such as the adhesive bonding of two mechanical components with large displacements and rotations [3] or bushing elements in automotive suspensions [6]. Objectivity and path independence are maintained. Due to the geometrically exact formulation, stability analyses that take the flexible connection into account can be performed. Due to the inertia terms considered, the coupling element can be taken into account in modal analyses. In this work, we focus on the coupling of nodes with three directors. Consequently, rigid bodies and geometrically exact beams can be coupled. An extension for nodes with one director, e.g. solid-degenerate shells, can be easily implemented.

**Fig. 1:**
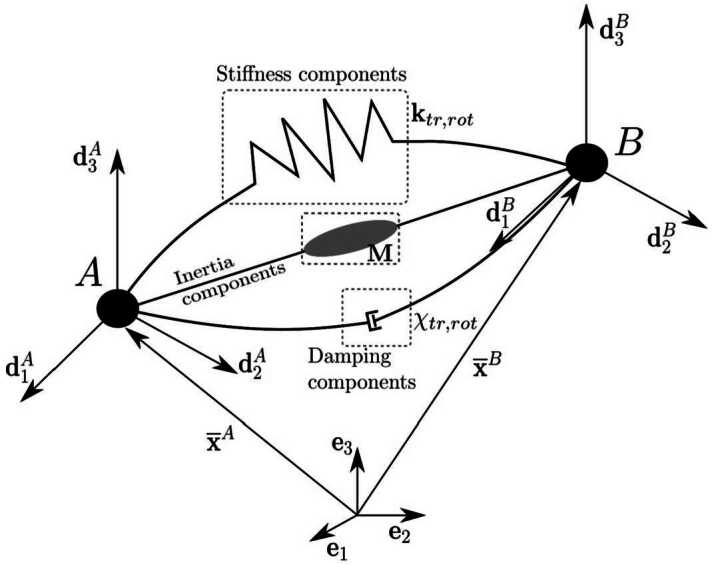
Schematic representation of the coupling element connecting two nodes

Figure 1 illustrates schematically the coupling element employed between two nodes $A$ and $B$. The Euclidean standard basis $\mathbf{e}_{i}$, the position vectors $\overline{\mathbf{x}}^{A}$, $\overline{\mathbf{x}}^{B}$ and the director triads $\mathbf{d}_{i}^{A}$ and $\mathbf{d}_{i}^{B}$ are shown. Due to the director-based formulation, generalised coordinates and generalised velocities are used. The generalised coordinates consist of a vector to a reference point $\mathbf{\overline{x}}$ belonging to the respective canonical model and the orthonormal directors $\mathbf{d}_{i}$ at that reference position

12 $$\begin{aligned} \mathbf{q}(t) &= [\mathbf{\overline{x}}(t),\mathbf{d}_{1}(t), \mathbf{d}_{2}(t),\mathbf{d}_{3}(t)]^{\text{T}} \in \mathbb{R}^{12 \times 1}. \end{aligned}$$

The components of the generalised coordinates are shown in Fig. 1. The superscripts $(\cdot )^{A}$ and $(\cdot )^{B}$ indicate which node the quantity refers to.

The velocity of the reference point is given by $\mathbf{\overline{v}}$. The velocity of the director triad is denoted by $\mathbf{w}_{i}$, leading to the vector of generalised velocities.

13 $$\begin{aligned} \mathbf{s}(t) &= [\mathbf{\overline{v}}(t),\mathbf{w}_{1}(t), \mathbf{w}_{2}(t),\mathbf{w}_{3}(t)]^{\text{T}} \in \mathbb{R}^{12 \times 1}. \end{aligned}$$

### Derivation and linearisation of the internal forces

In this contribution, we introduce a coupling element with a formulation that is consistent with that of the geometrically exact beam. This ensures objectivity and allows geometrically exact kinematics to be mapped.

We propose an objective strain measure inspired by the Green–Lagrange strain tensor. It is important to note that other deformation measures can also be used, provided that they satisfy the necessary requirements. As discussed in [27, 28, 30, 31], the Green–Lagrange strain tensor can be decomposed into two work-conjugate strain vectors of axial strains and curvatures. This is also applied to the deformation measure of the coupling element, leading to

14 $$\begin{aligned} \Gamma _{i} &= \langle \mathbf{A}(\mathbf{d}_{i}(t)) , \mathbf{D}( \mathbf{x}(t)) \rangle - \langle \mathbf{A}(\mathbf{d}_{i}(t_{0})), \mathbf{D}(\mathbf{x}(t_{0})) \rangle , \end{aligned}$$

15 $$\begin{aligned} \Omega _{i} &= \frac{1}{2}\varepsilon _{ijk}[\langle \mathbf{A}( \mathbf{d}_{k}(t)) , \mathbf{D}(\mathbf{d}_{j}(t))\rangle - \langle \mathbf{A}(\mathbf{d}_{k}(t_{0})), \mathbf{D}(\mathbf{d}_{j}(t_{0})) \rangle ]. \end{aligned}$$

Here, $\Gamma _{i}$ denotes the axial strains, $\Omega _{i}$ denotes the curvatures and $t_{0} = t(0)$. $\varepsilon _{ijk}$ is the permutation symbol. $\mathbf{A}(\cdot )$ and $\mathbf{D}(\cdot )$ symbolise an average and a discrete differential operator, respectively, defined as

16 $$\begin{aligned} \mathbf{A}(\mathbf{d}_{i}) = \frac{\mathbf{d}_{i}^{A}+\mathbf{d}_{i}^{B}}{2}, \quad \mathbf{D}( \overline{\mathbf{x}}) = \frac{\overline{\mathbf{x}}^{B}-\overline{\mathbf{x}}^{A}}{\eta}. \end{aligned}$$

The superscripts $(\cdot )^{A}$ and $(\cdot )^{B}$ indicate the nodes between which the coupling element is implemented. If we substitute the relationships defined in Equation (16) into Equations (14) and (15), we obtain

17 $$\begin{aligned} \boldsymbol{\Gamma}&= \frac{1}{2\eta}\left [ { \textstyle\begin{array}{c} \langle \overline{\mathbf{x}}^{B,t}-\overline{\mathbf{x}}^{A,t}, \mathbf{d}_{1}^{A,t}+\mathbf{d}_{1}^{B,t}\rangle -\langle \overline{\mathbf{x}}^{B,0}-\overline{\mathbf{x}}^{A,0},\mathbf{d}_{1}^{A,0}+ \mathbf{d}_{1}^{B,0} \rangle \\ \langle \overline{\mathbf{x}}^{B,t}-\overline{\mathbf{x}}^{A,t}, \mathbf{d}_{2}^{A,t}+\mathbf{d}_{2}^{B,t}\rangle -\langle \overline{\mathbf{x}}^{B,0}-\overline{\mathbf{x}}^{A,0},\mathbf{d}_{2}^{A,0}+ \mathbf{d}_{2}^{B,0}\rangle \\ \langle \overline{\mathbf{x}}^{B,t}-\overline{\mathbf{x}}^{A,t}, \mathbf{d}_{3}^{A,t}+\mathbf{d}_{3}^{B,t}\rangle -\langle \overline{\mathbf{x}}^{B,0}-\overline{\mathbf{x}}^{A,0},\mathbf{d}_{3}^{A,0}+ \mathbf{d}_{3}^{B,0}\rangle \end{array}\displaystyle } \right ], \end{aligned}$$

18 $$\begin{aligned} \boldsymbol{\Omega} &= \frac{1}{2\eta} \left [ { \textstyle\begin{array}{c} \langle \mathbf{d}_{2}^{A,t},\mathbf{d}_{3}^{B,t}\rangle -\langle \mathbf{d}_{3}^{A,t},\mathbf{d}_{2}^{B,t}\rangle - \langle \mathbf{d}_{2}^{A,0}, \mathbf{d}_{3}^{B,0}\rangle +\langle \mathbf{d}_{3}^{A,0},\mathbf{d}_{2}^{B,0} \rangle \\ \langle \mathbf{d}_{3}^{A,t},\mathbf{d}_{1}^{B,t}\rangle -\langle \mathbf{d}_{1}^{A,t},\mathbf{d}_{3}^{B,t}\rangle - \langle \mathbf{d}_{3}^{A,0}, \mathbf{d}_{1}^{B,0}\rangle +\langle \mathbf{d}_{1}^{A,0},\mathbf{d}_{3}^{B,0} \rangle \\ \langle \mathbf{d}_{1}^{A,t},\mathbf{d}_{2}^{B,t}\rangle -\langle \mathbf{d}_{2}^{A,t},\mathbf{d}_{1}^{B,t}\rangle - \langle \mathbf{d}_{1}^{A,0}, \mathbf{d}_{2}^{B,0}\rangle +\langle \mathbf{d}_{2}^{A,0},\mathbf{d}_{1}^{B,0} \rangle \end{array}\displaystyle } \right ]. \end{aligned}$$

The superscripts $(\cdot )^{t}$ and $(\cdot )^{0}$ indicate the time instant $t$ or the initial time $t=t_{0}$, respectively. The parameter $\eta $ is part of the definition of the chosen objective deformation tensor. The unit corresponds to a length. For a general discrete element, it holds $\eta = 1\text{ m}$. In principle, the parameter can be chosen arbitrarily but influences the deformation tensor. According to Equations (17) and (18), the coupling element behaves stiffer for $\eta > 1$ and softer for $\eta < 1$.

Equations (17) and (18) are summarised in a deformation vector $\mathbf{U}$, defined as

19 $$\begin{aligned} \mathbf{U}= \left [ { \textstyle\begin{array}{c} \boldsymbol{\Gamma} \\ \boldsymbol{\Omega} \end{array}\displaystyle } \right ] \in \mathbb{R}^{6\times 1}. \end{aligned}$$

As these are work-conjugate strain components, the axial and transverse shear forces $\mathbf{F}\in \mathbb{R}^{3\times 1}$ as well as the torsional and bending moments $\mathbf{M}\in \mathbb{R}^{3\times 1}$ can be obtained by deriving the elastic potential $W(\boldsymbol{\Gamma}, \boldsymbol{\Omega})$ with respect to the strain components

20 $$\begin{aligned} \mathbf{F} = \frac{\partial W_{int}}{\partial \boldsymbol{\Gamma}} \quad \text{and} \quad \mathbf{M} = \frac{\partial W_{int}}{\partial \boldsymbol{\Omega}} . \end{aligned}$$

Note that $\mathbf{F}$ refers hereafter to the force components and not to the tangent map.

The coupling element allows us to employ a fully populated symmetric elasticity matrix $\mathbf{C}$

21 $$C=\left[\begin{array}{cccccc} c_{11} & c_{12} & c_{13} & c_{14} & c_{15} & c_{16}\\ c_{21} & c_{22} & c_{23} & c_{24} & c_{25} & c_{26}\\ c_{31} & c_{32} & c_{33} & c_{34} & c_{35} & c_{36}\\ c_{41} & c_{42} & c_{43} & c_{44} & c_{45} & c_{46}\\ c_{51} & c_{52} & c_{53} & c_{54} & c_{55} & c_{56}\\ c_{61} & c_{62} & c_{63} & c_{64} & c_{65} & c_{66} \end{array}\right]=\left[\begin{array}{cc} C_{\Gamma \Gamma } & C_{\Gamma \Omega }\\ C_{\Omega \Gamma } & C_{\Omega \Omega } \end{array}\right].$$

The subscripts $\boldsymbol{\Gamma}$ and $\boldsymbol{\Omega}$ indicate the axial and transverse shear forces and the torsional and bending moments corresponding to the entries, respectively.

We subsequently derive the internal forces and moments caused by the elasticity of the coupling element. Therefore, we consider the variation of the strain energy, according to the principle of virtual work, as being

22 $$\begin{aligned} \delta W_{int} = \Bigl\langle \delta \boldsymbol{\Gamma}^{\text{T}}, \mathbf{F} \Bigr\rangle + \Bigl\langle \delta \boldsymbol{\Omega}^{ \text{T}}, \mathbf{M} \Bigr\rangle \end{aligned}$$

with the admissible variation $\delta (\cdot )$. This leads to

23 $$\begin{aligned} \delta W_{int}= \delta \mathbf{q}^{\text{T}} \left [ { \textstyle\begin{array}{c@{\quad}c} (\frac{\partial \boldsymbol{\Gamma}}{\partial \mathbf{q}})^{\text{T}} & (\frac{\partial \boldsymbol{\Omega}}{\partial \mathbf{q}})^{\text{T}} \end{array}\displaystyle } \right ] \left [ { \textstyle\begin{array}{c} \mathbf{F} \\ \mathbf{M} \end{array}\displaystyle } \right ] = \delta \mathbf{q}^{\text{T}} \mathbf{B}^{\text{T}} \mathbf{N}. \end{aligned}$$

$\mathbf{B}^{\text{T}} \in \mathbb{R}^{24\times 6}$ is a differential operator, while $\mathbf{N}\in \mathbb{R}^{6\times 1}$ is the combined vector of forces and moments

24 $$\begin{aligned} \mathbf{N} = \mathbf{C}\mathbf{U} = \mathbf{C}(\mathbf{B}^{\text{T}} \mathbf{q}). \end{aligned}$$

Carrying out the derivations introduced by the variation in Equation (23), it follows that for differential operator $\mathbf{B}^{\text{T}}$

25 $$\begin{aligned} \mathbf{B}^{\text{T}}=\frac{1}{2\eta} \left [ { \textstyle\begin{array}{c@{\quad}c@{\quad}c@{\quad}c@{\quad}c@{\quad}c} -(\mathbf{d}_{1}^{A}+ \mathbf{d}_{1}^{B}) & -(\mathbf{d}_{2}^{A}+ \mathbf{d}_{2}^{B})&-(\mathbf{d}_{3}^{A}+\mathbf{d}_{3}^{B}) & \mathbf{0} & \mathbf{0} & \mathbf{0} \\ (\mathbf{x}^{B}-\mathbf{x}^{A}) & \mathbf{0} & \mathbf{0} &\mathbf{0} & -\mathbf{d}_{3}^{B} & \mathbf{d}_{2}^{B} \\ \mathbf{0} &(\mathbf{x}^{B}-\mathbf{x}^{A}) & \mathbf{0} &\mathbf{d}_{3}^{B} & \mathbf{0} & -\mathbf{d}_{1}^{B} \\ \mathbf{0} & \mathbf{0} & (\mathbf{x}^{B}-\mathbf{x}^{A}) & - \mathbf{d}_{2}^{B} & \mathbf{d}_{1}^{B} & \mathbf{0} \\ (\mathbf{d}_{1}^{A}+\mathbf{d}_{1}^{B}) & (\mathbf{d}_{2}^{A}+ \mathbf{d}_{2}^{B}) &(\mathbf{d}_{3}^{A}+\mathbf{d}_{3}^{B}) & \mathbf{0} & \mathbf{0} & \mathbf{0} \\ (\mathbf{x}^{B}-\mathbf{x}^{A}) & \mathbf{0} & \mathbf{0} & \mathbf{0} & \mathbf{d}_{3}^{A} & -\mathbf{d}_{2}^{A} \\ \mathbf{0} &(\mathbf{x}^{B}-\mathbf{x}^{A}) & \mathbf{0} & - \mathbf{d}_{3}^{A} & \mathbf{0} & \mathbf{d}_{1}^{A} \\ \mathbf{0}& \mathbf{0}& (\mathbf{x}^{B}-\mathbf{x}^{A})&\mathbf{d}_{2}^{A}&- \mathbf{d}_{1}^{A}& \mathbf{0} \end{array}\displaystyle } \right ]. \end{aligned}$$

According to the time integration scheme presented in Sect. 2, Equation (25) is evaluated at the temporal midpoint. The discrete elastic force vector $\mathbf{f}^{\text{int}} \in \mathbb{R}^{24\times 1}$ is given as

26 $$\begin{aligned} \mathbf{f}^{\text{int}} = \mathbf{B}^{\text{T}}\mathbf{N}. \end{aligned}$$

The term representing the discrete internal forces of the coupling element is added to the discrete internal forces of the overall system in Equation (8). To apply Newton’s method in order to solve the nonlinear governing equation, we have to linearise the added discrete elastic forces, according to Equation (9). Respecting the product rule, we obtain

27 $$\begin{aligned} \Delta _{G} \delta W = \Delta _{G}(\delta \mathbf{q}^{\text{T}} \mathbf{B}^{\text{T}} \mathbf{N}) =\delta \mathbf{q}^{\text{T}}\Delta _{G}( \mathbf{f}^{\text{int}}). \end{aligned}$$

As $\mathbf{B}^{\text{T}}(\mathbf{q})$ and $\mathbf{N}(\mathbf{q})$ depend on the generalised coordinates $\mathbf{q}$, again the product rule is applied

28 $$\begin{aligned} \Delta _{G}(\mathbf{f}^{\text{int}})=\Delta _{G}(\mathbf{B}^{\text{T}} \mathbf{N}) =\Biggl(\left . \underbrace{\frac{\partial}{\partial \mathbf{q}}(\mathbf{B}^{\text{T}}\mathbf{C}\mathbf{B}^{\text{T}}\mathbf{q})}_{ \text{I}}\right |_{\mathbf{q}=\text{const.}}+ \left . \underbrace{\frac{\partial}{\partial \mathbf{q}}(\mathbf{B}^{\text{T}}\mathbf{N})}_{ \text{II}}\right |_{\mathbf{N}=\text{const.}}\Biggr)\Delta _{k} \mathbf{q}. \end{aligned}$$

After this, the two terms are linearised separately. The linearisation of the first term yields

29 $$\begin{aligned} \Delta _{G} \mathbf{N} &= \frac{\partial}{\partial \mathbf{q}}\Bigr( \mathbf{C}\mathbf{B}^{\text{T}}\mathbf{q}\Bigl) \Delta _{k}\mathbf{q} = \Biggl(\left . \frac{\partial}{\partial \mathbf{q}}\Bigr(\mathbf{C} \mathbf{B}^{\text{T}}\mathbf{q}\Bigl)\right |_{\mathbf{q = const.}} + \mathbf{C}\mathbf{B}^{\text{T}}\Biggr)\Delta _{k} \mathbf{q} \\ &= \Bigl(\mathbf{C}\mathbf{B}^{\text{T}}\Bigr)\Delta _{k}\mathbf{q}, \end{aligned}$$

with the given elasticity matrix $\mathbf{C}\in \mathbb{R}^{6\times 6}$ of the coupling element in Voigt notation. The linearisation of the second term of Equation (28) leads to

30 $$\begin{aligned} \left .\frac{\partial}{\partial \mathbf{q}}(\mathbf{B}^{\text{T}} \mathbf{N})\right |_{\mathbf{N}=\text{const.}}\Delta \mathbf{q} = \mathbf{K}_{\text{stress}}\Delta _{k}\mathbf{q}, \end{aligned}$$

with

31 $$\begin{aligned} \mathbf{K}_{\text{stress}} = \frac{1}{2\eta} \left [ { \textstyle\begin{array}{c@{\quad}c@{\quad}c@{\quad}c@{\quad}c@{\quad}c@{\quad}c@{\quad}c} \mathbf{0} & -F_{1}\mathbf{I} & -F_{2}\mathbf{I} & -F_{3}\mathbf{I} & \mathbf{0} & -F_{1}\mathbf{I} & -F_{2}\mathbf{I} & -F_{3}\mathbf{I} \\ -F_{1}\mathbf{I} & \mathbf{0}& \mathbf{0} & \mathbf{0} & F_{1} \mathbf{I} & \mathbf{0} & M_{3}\mathbf{I} & - M_{2}\mathbf{I} \\ -F_{2}\mathbf{I} & \mathbf{0} & \mathbf{0} & \mathbf{0} & F_{2} \mathbf{I} & -M_{3}\mathbf{I} & \mathbf{0} & M_{1} \mathbf{I} \\ -F_{3} \mathbf{I} & \mathbf{0} & \mathbf{0} & \mathbf{0} & F_{3} \mathbf{I}& M_{2}\mathbf{I} & -M_{1}\mathbf{I} & \mathbf{0} \\ \mathbf{0} & F_{1}\mathbf{I} & F_{2}\mathbf{I} & F_{3}\mathbf{I} & \mathbf{0} & F_{1}\mathbf{I} & F_{2}\mathbf{I} & F_{3}\mathbf{I} \\ -F_{1}\mathbf{I} &\mathbf{0} & -M_{3}\mathbf{I} & M_{2}\mathbf{I}& F_{1} \mathbf{I} & \mathbf{0} & \mathbf{0} & \mathbf{0} \\ -F_{2}\mathbf{I} &M_{3}\mathbf{I} & \mathbf{0} & - M_{1} \mathbf{I} & F_{2} \mathbf{I} & \mathbf{0} & \mathbf{0} & \mathbf{0} \\ -F_{3} \mathbf{I} & -M_{2}\mathbf{I} & M_{1}\mathbf{I} & \mathbf{0} & F_{3} \mathbf{I} & \mathbf{0} & \mathbf{0} & \mathbf{0} \end{array}\displaystyle } \right ] . \end{aligned}$$

If we substitute the results of the derivation back into Equation (27), then we obtain

32 $$\begin{aligned} \Delta _{G}\delta W &= \mathbf{K}_{\text{int}} = \delta \mathbf{q} \bigl(\underbrace{\mathbf{B}^{\text{T}}\mathbf{C}\mathbf{B}}_{ \mathbf{K}_{m} }+\mathbf{K}_{\text{stress}} \bigr)\Delta _{k} \mathbf{q} \\ &= \delta \mathbf{q} \bigl(\mathbf{K}_{m} + \mathbf{K}_{\text{stress}} \bigr)\Delta _{k}\mathbf{q}. \end{aligned}$$

Here, $\mathbf{K}_{\text{stress}}$ represents the stress-dependent stiffness contribution and $\mathbf{K}_{m}$ is the material stiffness contribution.

### Derivation of the inertia forces

To introduce mass properties into the coupling element, we have chosen a formulation equivalent to an arbitrarily shaped continuum modelled as a lumped element. This formulation is not equivalent to adding masses to the coupled nodes or elements. There are two reasons for this. Firstly, the location of the added masses would be different from the location of the coupling element mass and therefore the dynamic behaviour would be affected. Secondly, the mass distribution in the coupling element is not taken into account. The mass matrix of an arbitrarily shaped continuum can be derived as follows. The kinetic energy is denoted by

33 $$\begin{aligned} T_{\text{ac}} = \frac{1}{2}\int _{\theta _{3}}\int _{\theta _{2}}\int _{ \theta _{1}}\bigl\langle \rho \mathbf{v}_{\text{ac}}^{\text{T}}, \mathbf{v}_{\text{ac}}\bigr\rangle \text{d}\theta _{1} \text{d}\theta _{2} \text{d}\theta _{3}, \end{aligned}$$

with $\theta _{i}$ the director coordinates to a material point belonging to the rigid body.

We write the velocity in matrix notation

34 $$\begin{aligned} \mathbf{v}_{\text{ac}} = \left [ { \textstyle\begin{array}{c@{\quad}c@{\quad}c@{\quad}c} \mathbf{I} & \theta _{1}\mathbf{I} &\theta _{2}\mathbf{I} & \theta _{3} \mathbf{I} \end{array}\displaystyle } \right ] \left [ { \textstyle\begin{array}{c} \overline{\mathbf{v}} \\ \mathbf{w}_{1} \\ \mathbf{w}_{2} \\ \mathbf{w}_{3} \end{array}\displaystyle } \right ] = \boldsymbol{\Theta}^{\text{T}}\mathbf{s}_{\text{ac}} \end{aligned}$$

and obtain the constant mass matrix of the continuous element

35 $$\begin{aligned} \mathbf{M}_{\text{ac}} = \int _{\theta _{3}}\int _{\theta _{2}}\int _{ \theta _{1}} \boldsymbol{\Theta} ^{\text{T}}\rho \boldsymbol{\Theta} \text{d}\theta _{1} \text{d}\theta _{2} \text{d}\theta _{3} = \int _{ \mathcal{B}_{0}} \boldsymbol{\Theta}^{\text{T}} \rho \boldsymbol{\Theta} dV. \end{aligned}$$

This mass is assumed to be located between the connected nodes $A$ and $B$, as shown schematically in Fig. 1. It remains at the centre of the nodes considered at all times and is not weighted along the length of the element.

To derive the inertia forces acting on the connected nodes, we need the mass matrix of the coupling element with equivalent properties as just demonstrated for the arbitrarily shaped continuum. It can be obtained by writing down the kinetic energy of the coupling element. All quantities without subscripts refer to the coupling element.

36 $$\begin{aligned} T = \frac{1}{2}\Bigl\langle \mathbf{s}^{AB},\mathbf{M}_{\text{ac}} \mathbf{s}^{AB}\Bigr\rangle . \end{aligned}$$

$\mathbf{s}^{AB}$ denotes the velocity of the midpoint between the nodes. Consequently, it is determined as follows:

37 $$\begin{aligned} \mathbf{s}^{AB} = \frac{\mathbf{s}^{A}+\mathbf{s}^{B}}{2}, \end{aligned}$$

using the generalised velocities of $\mathbf{s}^{A}$ and $\mathbf{s}^{B}$ of the respective nodes. Substituting the velocity $\mathbf{s}^{AB}$ into Equation (36), allows us to determine the mass matrix $\mathbf{M}_{\text{ce}}$ of the coupling element.

38 $$\begin{aligned} T &=\frac{1}{2}\Bigl\langle \Bigl( \frac{\mathbf{s}^{A}+\mathbf{s}^{B}}{2}\Bigr), \mathbf{M}_{\text{ac}} \Bigl(\frac{\mathbf{s}^{A}+\mathbf{s}^{B}}{2}\Bigr)\Bigr\rangle \\ &= \frac{1}{2}\Bigl\langle \left [ { \textstyle\begin{array}{c@{\quad}c} \mathbf{s}^{A^{\text{T}}} & \mathbf{s}^{B^{\text{T}}} \end{array}\displaystyle } \right ],\mathbf{M}_{\text{ce}} \left [ { \textstyle\begin{array}{c} \mathbf{s}^{A} \\ \mathbf{s}^{B} \end{array}\displaystyle } \right ]\Bigr\rangle , \end{aligned}$$

with

39 $$\begin{aligned} \mathbf{M}_{\text{ce}}& = \frac{1}{4}\left [ { \textstyle\begin{array}{c@{\quad}c} \mathbf{M}_{\text{ac}} & \mathbf{M}_{\text{ac}} \\ \mathbf{M}_{\text{ac}} & \mathbf{M}_{\text{ac}} \end{array}\displaystyle } \right ]. \end{aligned}$$

The accelerations of the nodes in Equation (7) are determined using the discrete time derivative of the nodes’ generalised velocities

40 $$\begin{aligned} \dot{\mathbf{s}}^{A}_{t_{n+\frac{1}{2}}} \approx \frac{\mathbf{s}^{A}_{t_{n+1}}-\mathbf{s}^{A}_{t_{n}}}{\Delta t}, \quad \dot{\mathbf{s}}^{B}_{t_{n+\frac{1}{2}}} \approx \frac{\mathbf{s}^{B}_{t_{n+1}}-\mathbf{s}^{B}_{t_{n}}}{\Delta t}. \end{aligned}$$

For the time derivative of the momentum, the inertia forces acting on the two connected nodes are as follows:

41 $$\begin{aligned} \dot{\mathbf{l}}(\mathbf{s}_{t_{n+\frac{1}{2}}})=\mathbf{M}_{ \text{ce}}\dot{\mathbf{s}}^{AB}_{t_{n+\frac{1}{2}}}=\mathbf{M}_{ \text{ce}} \left [ { \textstyle\begin{array}{c} \dot{\mathbf{s}}^{A}_{t_{n+\frac{1}{2}}} \\ \dot{\mathbf{s}}^{B}_{t_{n+\frac{1}{2}}} \end{array}\displaystyle } \right ]. \end{aligned}$$

The mixed formulation and the use of the velocity in the inertia term make it necessary to establish the linear equilibrium of momentum to ensure that $\dot{\mathbf{q}} = \mathbf{s}$ at any time instant.

42 $$\begin{aligned} \mathbf{l}(\mathbf{q})-\mathbf{l}(\mathbf{s})=\mathbf{M}_{\text{ce}} \bigl( \frac{\mathbf{q}^{AB}_{t_{n+1}}-\mathbf{q}^{AB}_{t_{n}}}{\Delta t}- \frac{\mathbf{s}^{AB}_{t_{n+1}}+\mathbf{s}^{AB}_{t_{n}}}{2}\bigr). \end{aligned}$$

As with the internal forces arising due to the elasticity of the coupling element, the inertia forces related to the mass are also linearised. The derivative of $\dot{\mathbf{l}}(\mathbf{s})$ with respect to $\mathbf{q}$ becomes zero.

43 $$\begin{aligned} \frac{\partial}{\partial \mathbf{q}^{AB}_{t_{n+1}}}\Bigl( \mathbf{M}_{ \text{ce}}\dot{\mathbf{s}}^{AB}_{t_{n+\frac{1}{2}}}\Bigr) = 0. \end{aligned}$$

Because the term depends on the generalised velocities to provide the acceleration $\dot{\mathbf{s}}^{AB}_{t_{n+\frac{1}{2}}} \approx \frac{\mathbf{s}^{AB}_{t_{n+1}}-\mathbf{s}^{AB}_{t_{n}}}{\Delta t}$, linearisation with respect to the generalised velocity is needed. This term reads

44 $$\begin{aligned} \mathbf{K}_{qs}&=\frac{\partial}{\partial \mathbf{s}^{AB}_{t_{n+1}}} \Bigl( \mathbf{M}_{\text{ce}}\dot{\mathbf{s}}^{AB}_{t_{n+\frac{1}{2}}} \Bigr) \\ &= \frac{\partial}{\partial \mathbf{s}^{AB}_{t_{n+1}}} \Bigl( \mathbf{M}_{\text{ce}}\Bigl( \frac{\mathbf{s}^{AB}_{t_{n+1}}-\mathbf{s}^{AB}_{t_{n}}}{\Delta t} \Bigr)\Bigr) \\ &=\frac{1}{\Delta t}\mathbf{M}_{\text{ce}}. \end{aligned}$$

The equivalence of momentum is derived accordingly with respect to $\mathbf{q}$ and $\mathbf{s}$:

45 $$\begin{aligned} \mathbf{K}_{sq}&=\frac{\partial}{\partial \mathbf{q}^{AB}_{t_{n+1}}} \Bigl(\mathbf{M}_{\text{ce}}\Bigl( \frac{\mathbf{q}^{AB}_{t_{n+1}}-\mathbf{q}^{AB}_{t_{n+}}}{\Delta t}- \frac{\mathbf{s}^{AB}_{t_{n+1}}+\mathbf{s}^{AB}_{t_{n}}}{2} \Bigr) \Bigr) \\ &= \frac{1}{\Delta t}\mathbf{M}_{\text{ce}}, \end{aligned}$$

46 $$\begin{aligned} \mathbf{K}_{ss}&=\frac{\partial}{\partial \mathbf{s}^{AB}_{t_{n+1}}} \Bigl(\mathbf{M}_{\text{ce}}\Bigl( \frac{\mathbf{q}^{AB}_{t_{n+1}}-\mathbf{q}^{AB}_{t_{n}}}{\Delta t}- \frac{\mathbf{s}^{AB}_{t_{n+1}}+\mathbf{s}^{AB}_{t_{n}}}{2} \Bigr) \Bigr) \\ &=- \frac{1}{2}\mathbf{M}_{\text{ce}}. \end{aligned}$$

### Derivation of the damping forces

It remains to introduce a damping formulation into the coupling element to dissipate numerically nonphysical high frequencies and to model physical damping properties of flexible couplings. We chose a damping formulation according to [19, 28].

The formulation chosen in the spatially and temporally discrete governing equation (7) allows the use of first-order dissipation functions. Higher-order dissipation functions are possible but are beyond the scope of this paper. To demonstrate that different first-order dissipation functions can be used, we implement two different dissipation functions. In future work, these dissipation functions can be adapted to suit other physical problems that need to be addressed, such as specific material damping properties.

The applied stress/strain-dependent damping is added as a nonconservative term to the internal forces

47 $$\begin{aligned} \mathbf{f}^{\text{int}} = \mathbf{f}^{\text{int}}_{\text{conservative}} + \mathbf{f}^{\text{int}}_{\text{non-conservative}}. \end{aligned}$$

#### First-order dissipation scheme

The dissipation function proposed by Armero et al. [28] is employed. Gebhardt et al. [19] also developed a similar kind of damping algorithm but followed a derivation strategy based on the *average-vector-field*. They added perturbations to the discretised equation of motion, which led to conservation or dissipation properties. They proposed both a stress/strain-based damping algorithm and a velocity-based algorithm. In the present work, we focus on the stress/strain-based damping forces and moments andin a second step modify the equation to demonstrate the possibility of implementing different dissipation functions using the example of a strain-rate/stress-rate dependent dissipation function.

Without discussing the particulars, we refer the reader to the aforementioned literature concerning the derivation of the correlation between deformation and damping forces and moments. This formulation naturally maintains the framework’s objectivity. The forces and moments are determined as follows:

48 $$\begin{aligned} \mathbf{F}_{\text{diss}}&= \frac{\textit{D}_{\Gamma}}{\|\boldsymbol{\Gamma}_{t_{n+1}}-\boldsymbol{\Gamma}_{t_{n}}\|_{\mathbf{C}_{\Gamma}}} \cdot \frac{\mathbf{C}_{\Gamma}(\boldsymbol{\Gamma}_{t_{n+1}}-\boldsymbol{\Gamma}_{t_{n}})}{\|\boldsymbol{\Gamma}_{t_{n+1}}-\boldsymbol{\Gamma}_{t_{n}}\|_{\mathbf{C}_{\Gamma}}}, \end{aligned}$$

49 $$\begin{aligned} \mathbf{M}_{\text{diss}}&= \frac{\textit{D}_{\Omega}}{\|\boldsymbol{\Omega}_{t_{n+1}}-\boldsymbol{\Omega}_{t_{n}}\|_{\mathbf{C}_{\Omega}}} \cdot \frac{\mathbf{C}_{\Omega}(\boldsymbol{\Omega}_{t_{n+1}}-\boldsymbol{\Omega}_{t_{n}})}{\|\boldsymbol{\Omega}_{t_{n+1}}-\boldsymbol{\Omega}_{t_{n}}\|_{\mathbf{C}_{\Omega}}}. \end{aligned}$$

$\|\dots \|_{\mathbf{C}} = \sqrt{\langle (\dots )^{\text{T}}, \mathbf{C}\cdot (\dots )\rangle}$ denotes the weighted vector norm. $\mathbf{C}_{\Gamma}$ and $\mathbf{C}_{\Omega}$ are the elasticity matrices according to the deformations $\boldsymbol{\Gamma}$ and $\boldsymbol{\Omega}$, respectively. Analogous to the conservative forces and moments, we summarise the dissipative forces and moments in the vector $\mathbf{\mathbf{N}_{\text{diss}}}$

50 $$\begin{aligned} \mathbf{N}_{\text{diss}}= \left [ { \textstyle\begin{array}{c} \mathbf{F}_{\text{diss}} \\ \mathbf{M}_{\text{diss}} \end{array}\displaystyle } \right ]. \end{aligned}$$

It remains to define the scalar dissipation functions $\textit{D}_{\Gamma}$ and $\textit{D}_{\Omega}$. They are proposed as

51 $$\begin{aligned} \textit{D}_{\Gamma}&= \frac{1}{2}\chi _{\Gamma}\|\boldsymbol{\Gamma}_{t_{n+1}}- \boldsymbol{\Gamma}_{t_{n}}\|^{2}_{\mathbf{C}_{\Gamma}}, \end{aligned}$$

52 $$\begin{aligned} \textit{D}_{\Omega}&= \frac{1}{2}\chi _{\Omega}\|\boldsymbol{\Omega}_{t_{n+1}}- \boldsymbol{\Omega}_{t_{n}}\|^{2}_{\mathbf{C}_{\Omega}}. \end{aligned}$$

The dimensionless parameters $\chi _{\Gamma}$ and $\chi _{\Omega}$ allow to determine the scale of the dissipation forces and moments. They can be adapted by the user according to the model. This leads to the implemented terms for the discrete damping force and moment:

53 $$\begin{aligned} \mathbf{F}_{\text{diss}}&=\frac{1}{2}\chi _{\Gamma} \mathbf{C}_{\Gamma}( \boldsymbol{\Gamma}_{t_{n+1}}-\boldsymbol{\Gamma}_{t_{n}}), \end{aligned}$$

54 $$\begin{aligned} \mathbf{M}_{\text{diss}}&=\frac{1}{2}\chi _{\Omega} \mathbf{C}_{\Omega}( \boldsymbol{\Omega}_{t_{n+1}}-\boldsymbol{\Omega}_{t_{n}}). \end{aligned}$$

#### Modified first-order dissipation scheme

We demonstrate in the following that the formulation briefly summarised in Sect. 3.3.1 can encompass various dissipation functions. The parameters $\chi _{\Gamma}$ and $\chi _{\Omega}$ can be chosen arbitrarily to adapt the damping magnitude to the specific application. Therefore, it is appropriate to choose $\chi _{\Gamma }= \frac{\alpha _{\Gamma}}{\Delta t}$ and $\chi _{\Omega }= \frac{\alpha _{\Omega}}{\Delta t}$, which implies that the damping forces and moments are dependent on the deformation rate rather than on the absolute deformation. $\alpha _{\Gamma}$ and $\alpha _{\Omega}$ are again dimensionless parametrisation factors. This inspires the conversion of the numerical derivatives into analytical derivatives of the deformation measure.

55 $$\begin{aligned} \mathbf{F}_{\text{diss}}=\lim _{\Delta t \to 0}\frac{1}{2}\alpha _{ \Gamma}\mathbf{C}_{\Gamma} \frac{\boldsymbol{\Gamma}_{t_{n+1}}-\boldsymbol{\Gamma}_{t_{n}}}{\Delta t} \quad &\rightarrow \quad \mathbf{\tilde{F}}_{\text{diss}} = \frac{1}{2}\alpha _{\Gamma}\mathbf{C}_{\Gamma}\dot{\boldsymbol{\Gamma}}_{t_{n+ \frac{1}{2}}}, \end{aligned}$$

56 $$\begin{aligned} \mathbf{M}_{\text{diss}}=\lim _{\Delta t \to 0}\frac{1}{2}\alpha _{ \Omega}\mathbf{C}_{\Omega} \frac{\boldsymbol{\Omega}_{t_{n+1}}-\boldsymbol{\Omega}_{t_{n}}}{\Delta t} \quad &\rightarrow \quad \mathbf{\tilde{M}}_{\text{diss}} = \frac{1}{2}\alpha _{\Omega}\mathbf{C}_{\Omega}\dot{\boldsymbol{\Omega}}_{t_{n+ \frac{1}{2}}}. \end{aligned}$$

For the sake of completeness, a scalar dissipation function can also be identified for this purpose in accordance with the derivation from [19]. A minimum dissipative force is sought that satisfies the relationship (analogously for $\boldsymbol{\Omega}$) denoted in Equation (58)

57 $$\begin{aligned} &\frac{1}{2}\|\mathbf{F}_{\text{diss}}\|^{2}_{\mathbf{C}} \quad \rightarrow \quad \text{min}, \end{aligned}$$

58 $$\begin{aligned} &\langle \mathbf{F}_{\text{diss}}, \boldsymbol{\Gamma}_{t_{n+1}}- \boldsymbol{\Gamma}_{t_{n}}\rangle -\mathcal{D}(\boldsymbol{\Gamma}_{t_{n+1}}, \boldsymbol{\Gamma}_{t_{n}}) = 0. \end{aligned}$$

We modify this derivation so that the dissipation force depends on the deformation rate instead on the absolute deformation:

59 $$\begin{aligned} &\frac{1}{2}\|\mathbf{F}_{\text{diss}}\|^{2}_{\mathbf{C}} \quad \rightarrow \quad \text{min}, \end{aligned}$$

60 $$\begin{aligned} &\langle \mathbf{F}_{\text{diss}}, \dot{\boldsymbol{\Gamma}}_{t_{n+ \frac{1}{2}}}\rangle -\mathcal{D}(\dot{\boldsymbol{\Gamma}}_{t_{n+ \frac{1}{2}}}) = 0. \end{aligned}$$

Without repeating the derivation, this leads to the strain-rate dependent dissipation functions

61 $$\begin{aligned} \tilde{\mathcal{D}}_{\Gamma }&= \frac{1}{2}\alpha _{\dot{\Gamma}} \bigl\langle \mathbf{C}\dot{\boldsymbol{\Gamma}}_{t_{n+\frac{1}{2}}}, \dot{\boldsymbol{\Gamma}}_{t_{n+\frac{1}{2}}}\bigr\rangle , \end{aligned}$$

62 $$\begin{aligned} \tilde{\mathcal{D}}_{\Omega }&= \frac{1}{2}\alpha _{\dot{\Omega}} \bigr\langle \mathbf{C}\dot{\boldsymbol{\Omega}}_{t_{n+\frac{1}{2}}}, \dot{\boldsymbol{\Omega}}_{t_{n+\frac{1}{2}}}\bigr\rangle . \end{aligned}$$

The analytical time derivative of the deformation measure $\mathbf{u}$ is determined as

63 $$\begin{aligned} \dot{\mathbf{u}} &= \frac{\partial}{\partial t} \mathbf{u}= \left [ { \textstyle\begin{array}{c} \dot{\boldsymbol{\Gamma}} \\ \dot{\boldsymbol{\Omega}} \end{array}\displaystyle } \right ] \end{aligned}$$

64 $$\begin{aligned} &=\frac{1}{2\eta} \left [ { \textstyle\begin{array}{c} \mathbf{x}^{B}(\dot{\mathbf{d}}_{i}^{A}+\dot{\mathbf{d}}_{i}^{B}))+ \dot{\mathbf{x}}^{B}(\mathbf{d}_{i}^{A}+\mathbf{d}_{i}^{B}))- \mathbf{x}^{A}(\dot{\mathbf{d}}_{i}^{A}+\dot{\mathbf{d}}_{i}^{B}))- \dot{\mathbf{x}}^{A}(\mathbf{d}_{i}^{A}+\mathbf{d}_{i}^{B}) \\ \varepsilon _{ijk}(\dot{\mathbf{d}}_{j}^{A} \mathbf{d}_{k}^{B} + \dot{\mathbf{d}}_{k}^{B} \mathbf{d}_{j}^{A} - \dot{\mathbf{d}}_{k}^{A} \mathbf{d}_{j}^{B} - \dot{\mathbf{d}}_{j}^{B} \mathbf{d}_{k}^{A}) \end{array}\displaystyle } \right ] , \end{aligned}$$

using $\varepsilon _{ijk}$, the permutation symbol and $i,j,k = 1,2,3$. Again, to maintain the readability, we have not indicated the time instants. The deformation components are evaluated at the midpoint as

65 $$\begin{aligned} \mathbf{u} = \mathbf{u}_{t_{n+\frac{1}{2}}} \approx \frac{\mathbf{u}_{t_{n+1}}+\mathbf{u}_{t_{n}}}{2}, \quad \dot{\mathbf{u}}= \dot{\mathbf{u}}_{t_{n+\frac{1}{2}}} \approx \frac{\dot{\mathbf{u}}_{t_{n+1}}+\dot{\mathbf{u}}_{t_{n}}}{2}. \end{aligned}$$

Note that derivations of objective quantities defined in local coordinate systems may not necessarily be objective. As the selected deformation measure comprises quantities defined in the global coordinate system, their time derivatives are also objective.

The damping forces and moments for the first-order dissipation scheme are summarised in a vector

66 $$\begin{aligned} \mathbf{N}_{\text{diss}} = \frac{1}{2}\boldsymbol{\chi}\mathbf{C} \mathbf{u} = \frac{1}{2}\boldsymbol{\chi}\mathbf{C}(\mathbf{B}^{ \text{T}}\mathbf{q}), \end{aligned}$$

with

67 $$\begin{aligned} \boldsymbol{\chi} = \left [ { \textstyle\begin{array}{c@{\quad}c} \chi _{\Gamma }\mathbf{I} & 0 \\ 0 & \chi _{\Omega }\mathbf{I} \end{array}\displaystyle } \right ] \in \mathbb{R}^{6\times 6}. \end{aligned}$$

$\mathbf{I} \in \mathbb{R}^{3\times 3}$ denotes the identity matrix. For the linearisation, it follows consequently

68 $$\begin{aligned} \mathbf{K}_{\text{int,diss}} = \frac{\partial \mathbf{N}_{\text{diss}}}{\partial \mathbf{q}} = \frac{1}{2}\boldsymbol{\chi}\mathbf{C}\mathbf{B}^{\text{T}}. \end{aligned}$$

The same entries in the tangent operator can be used in the modified first-order dissipation scheme for sufficiently small $\Delta t$ as the expressions are equal for $\Delta t \to 0$.

## Applications

In this section, some application and benchmark examples are shown to illustrate the behaviour of the coupling element. The nonlinear mechanical model of the in-house multi-physical simulation software DeSiO is used. This is briefly presented in Sect. 2. This framework has recently been developed in the context of wind energy, especially to tackle the nonlinear behaviour of large offshore wind turbines.

In Sect. 4.1, a check of the coupling element formulation’s plausibility is presented. In Sect. 4.2, we illustrate the geometrically exact behaviour, a verification against ABAQUS for small displacements and a numerical indication of path independence. Since no geometrically exact spring elements can be used in ABAQUS, we verify the static behaviour of the coupling element on small displacements. Here, linear (ABAQUS) and nonlinear (coupling element) deformation measures show the same load-deformation behaviour. A transient and modal analysis is presented in Sect. 4.3 to illustrate the dissipation of high frequencies and the reduction of the total energy. Finally, in Sect. 4.4, we qualitatively demonstrate how the coupling element can be used to model the soil–structure interaction of a wind turbine. We show the influence of the flexible support on the natural frequencies of the complex structure.

### Plausibility check of the coupling element formulation in a transient analysis

The plausibility check shown here is only possible because the replaced beam element also uses a geometrically nonlinear Green–Lagrange deformation measure and additionally employs linear shape functions. Of course, nodes of for example a linear Euler–Bernoulli beam could also be connected, but in this case, the plausibility check would not be meaningful as the results would differ.

The deformation measure used for the coupling element is based on the Green–Lagrange strain tensor given in Equation (5). As shown in Equations (17) and (18), the strain tensor can be parameterised by the choice of $\eta $. To prove that the formulations are transferable to the geometrically exact beam, we use this parameter to obtain an equivalent formulation. Furthermore, we model an example application with the coupling element or the geometrically exact beam.

The parameter is $\eta = 1 \text{ m}$ for the coupling element. To obtain the geometrically exact beam formulation, we need to choose $\eta = l$, where $l$ is the length of the beam element, i.e.the distance between the connected nodes resulting from the FE discretisation. The resulting modification of the nonlinear governing equation is taken into account in the tangent operator as follows:

69 $$\begin{aligned} \mathbf{K}_{qq} = \frac{1}{2}\bigl( \mathbf{K}_{1} + \mathbf{K}_{2} + \mathbf{K}_{m} \bigr) \quad \rightarrow \quad \mathbf{K}_{qq, \text{mod}} = j \cdot w \cdot \mathbf{K}_{qq}, \end{aligned}$$

with the Jacobian $j = l/2$ and the weight $w=2$, both resulting from the Gaussian-quadrature as part of the FE formulation. Again, we refer to the description of the geometrically exact beam theory in the literature, e.g. [27]. As the inertia terms can not be reverted to the beam element formulation through parametrisation, we adopt the beam element formulation in this example.

**Fig. 2:**
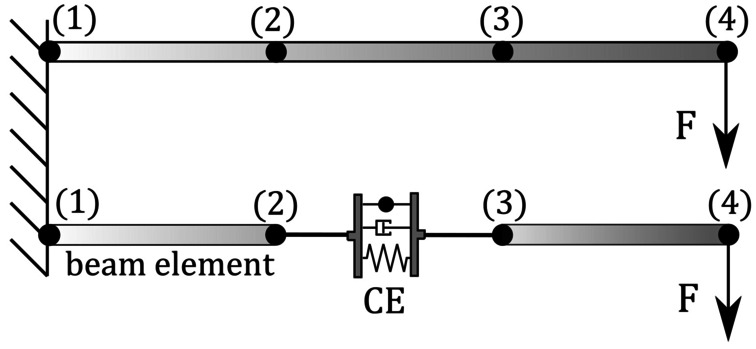
Beam/coupling element configuration used to compare the resulting tangent operator

The example is schematically illustrated in Fig. 2. As a benchmark example, we chose a horizontally orientated geometrically exact beam, with a length $L=3.0\text{ m}$. The beam is discretised into three elements, with $l=1.0\text{ m}$, using four nodes (1) to (4). The cross-section properties of the beam are equivalent to a steel beam with a rectangular geometry ($0.02 \text{ m} \times 0.02 \text{ m}$), with a mass density $\rho _{steel} = 7580 \frac{\text{kg}}{\text{m}^{3}}$, a Young’s modulus $\text{E}_{steel} = 210\text{ GPa}$ and a Poisson’s ratio $\nu _{steel} = 0.3$. Node (1) of the beam is clamped. A vertical force of $F = 50.0\text{ kN}$ is applied to the tip of the beam at node (4). The force is linearly increased within in the time interval $t = [0.0,2.0]\text{ s}$.

In a second model, the beam element between nodes (2) and (3) is removed. Instead, the coupling element replaces the geometrically exact beam element. It is parameterised with $\eta = l$ as described above to obtain the exact same formulation as the beam element. The material parameters are the same as for the beam element. The loading scenario is also identical. To compare the formulations, a transient analysis of the systems is performed. The total simulation time is $T = 5.0 \text{ s}$ with a time step size of $\Delta t = 1.0\cdot 10^{-2} \text{ s}$. Since the linearly applied forces are removed after $t = 2.0 \text{ s}$, the systems can oscillate freely. The following boundary and constraint conditions were applied: the clamping of node (1) is realised by preventing all displacements and rotations of the node. An internal constraint is used for each node (2), (3) and (4). This ensures that the directors continue to provide an orthonormal basis with constant length. No internal constraint is required for node (1) since the prevention of rotations and displacements is redundant.

**Fig. 3:**
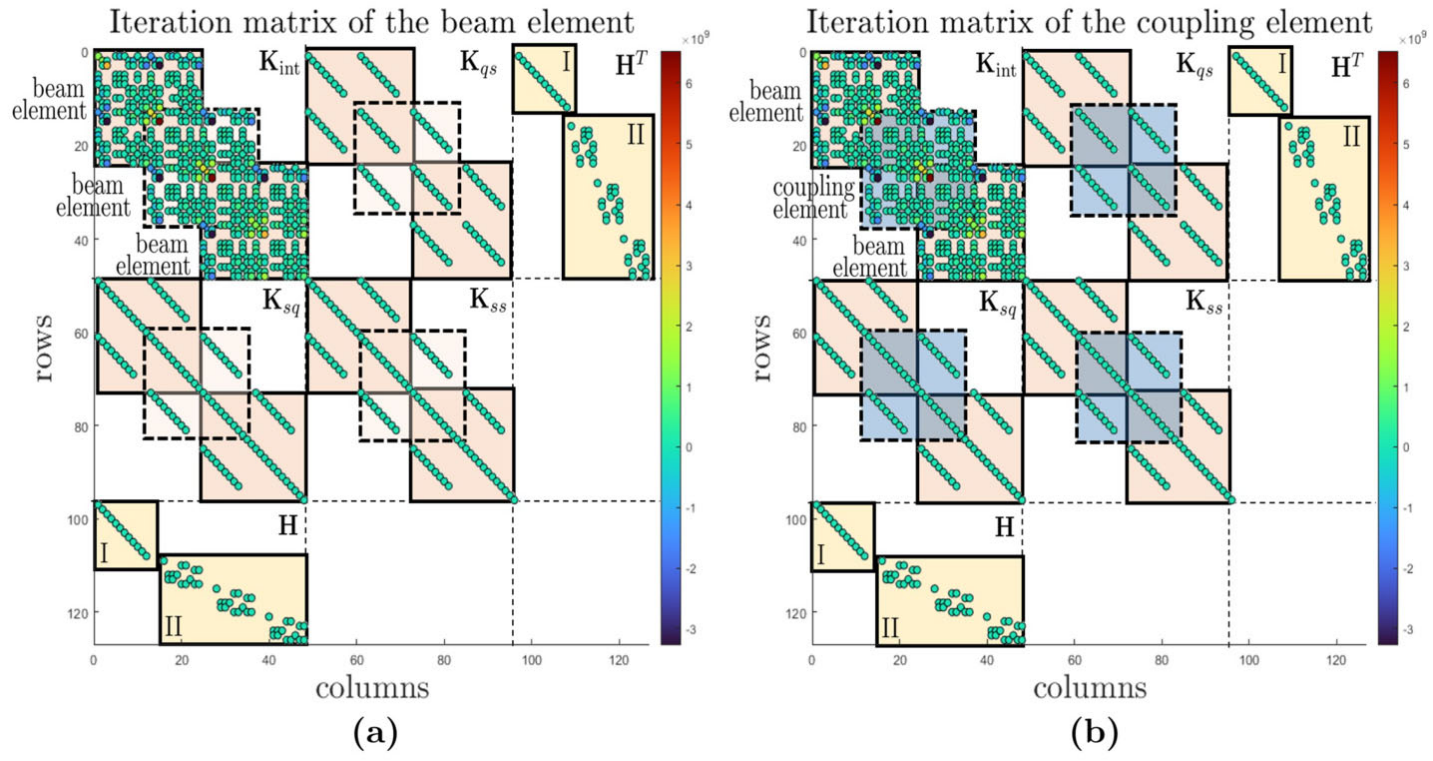
**(a)** Tangent operator for $n_{el} = 3$ beam elements at time $T=5.0\text{ s}$, orange: terms relating to beam elements, yellow: terms related to boundary and constraint conditions; **(b)** Tangent operator for $n_{el} = 2$ beam elements + $n_{ce} = 1$ coupling element at time $T=5.0\text{ s}$, orange: terms related to beam elements, blue: terms related to the coupling element, yellow: terms related to boundary and constraint conditions (Colour figure online)

As the formulations of the beam element and the coupling element are aligned through the parameterisation of the deformation measure of the coupling element, we expect that the iteration matrices will also be identical when solving the nonlinear governing equations. Accordingly, the residuals in Newton’s method and the displacement of the beam tip must also coincide. Figure 3 shows the tangent operator for the system consisting exclusively of geometrically exact beam elements 3 (a) and also for the system where a beam element has been replaced by the coupling element 3 (b) of time step 500, corresponding to the last simulated time step at $T = 5\text{ s}$. In Figs. 3 (a) and 3 (b), each dot represents a nonzero entry in the respective tangent operator. The value is indicated by its colour. Labels are added to the matrices to indicate which terms are contained within the fields bounded by the dotted lines. Additionally, coloured squares are drawn within these fields. The orange fields indicate that the entries originate from the beam formulation. The blue squares in Fig. 3 (b) indicate which fields are derived from the coupling element formulation. Terms from the boundary and constraint conditions are marked in yellow. The entries labelled “I” arise from the boundary condition that provides fixed support. Entries labelled “II” are the result of the internal constraints. Of course, the chosen beam/coupling element formulation does not affect the boundary and constraint conditions of the model. Figure 3 is intended to illustrate visually how the sparse tangent operator is composed and in which areas it is influenced by the coupling element.

Checking the values of the entries shows that not only the structure of the matrices, as already suggested by the visual comparison, but also the values are identical. This demonstrates that the formulation of the coupling element can be transformed into the formulation of the geometrically exact beam. Furthermore, this example serves as a plausibility check to ensure the correctness of the implementation. For this purpose, we also compare the norms of the residuals in Newton’s method and the displacement of the tip node (4) after a simulation time of $T=5.0\text{ s}$. As can be seen in Table 1, the convergence behaviour and the final displacement of node (4) are identical for the system consisting of beam elements and the system that substitutes a beam element with the coupling element. This shows that the same problem is solved and demonstrates the plausibility of the implementation. Furthermore, the quadratic convergence behaviour is evident.

**Table 1:** Comparison of the norm of the Newton residuals and the displacement of node (4) at the time instant $T=5.0\text{ s}$

	Beam	Beam + CE
Norm of Newton residuals	0.57	0.57
	0.17	0.17
	0.43⋅10^−2^	0.43⋅10^−2^
	0.11⋅10^−4^	0.11⋅10^−4^
	0.18⋅10^−8^	0.18⋅10^−8^
Final displacement node (4)	0.1921 m	0.1921 m

At this point we underline that the coupling element can be reverted into the beam element by the measures described above. This is possible because of the more general formulation of the coupling element. It has been shown that the stiffness properties can be reverted by suitable parameterisation of $\eta $. However, the inertia terms could not be obtained by parameterising the coupling element alone. Conversely, it is clear that the beam element cannot replace the coupling element. As long as strain/stress dependent dissipation is introduced into the system, the coupling element in its present form could also represent this. The modified dissipation function could, in principle, also be implemented in the beam element. However, it has been developed here for demonstration purposes.

### Static analyses

The coupling element employs a nonlinear deformation measure with geometrically exact behaviour. As discussed, it is therefore well suited to modelling large displacements and rotations of a coupling. For small displacements and rotations, the geometric nonlinearity can be neglected. Consequently, it is possible to verify the coupling element’s behaviour against an implementation of a linear deformation measure if the occurring displacements and rotations remain small. We consider a simple example of a beam, which is flexibly supported by the coupling element. A load at the tip of the beam induces a moment acting on the coupling element. Using that example, we first show the transition of linear to geometrically nonlinear behaviour in Sect. 4.2.1. In Sect. 4.2.2, we implement the same beam structure in ABAQUS where a set of linear spring elements is used to model the flexible mounting. The ABAQUS model is used to verify the behaviour for small rotations. In 4.2.3, we demonstrate the path independence numerically.

**Fig. 4:**
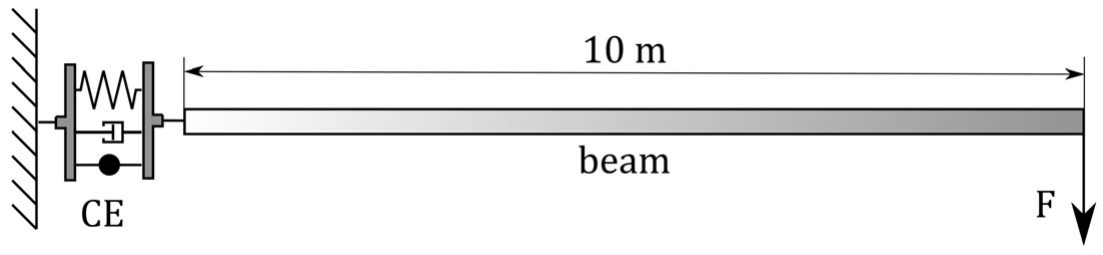
Schematic representation of a statically loaded beam, flexibly mounted to the environment

The system under consideration is schematically illustrated in Fig. 4. The beam has a length of $l = 10\text{ m}$. Its cross-section is square with a width of $0.1\text{ m}$. The material used is steel with $\rho _{steel} = 7580 \frac{\text{kg}}{\text{m}^{3}}$, $\text{E}_{steel} = 210 \text{ GPa}$ and $\nu _{steel}= 0.3$. The geometrically exact beam is discretised with $n_{el} = 20$ elements. A diagonal elasticity matrix is used for the coupling element ($c_{11}= c_{22}=c_{33}=2 \cdot 10^{6}\frac{\text{N}}{\text{m}}$, $c_{44}=c_{55}=c_{66}=5\cdot 10^{4} \frac{\text{Nm}}{\text{rad}}$), see Equation (21). The subsequently discussed static analyses are performed without considering self-weight.

#### Illustration of the geometrically exact mechanical behaviour

**Fig. 5:**
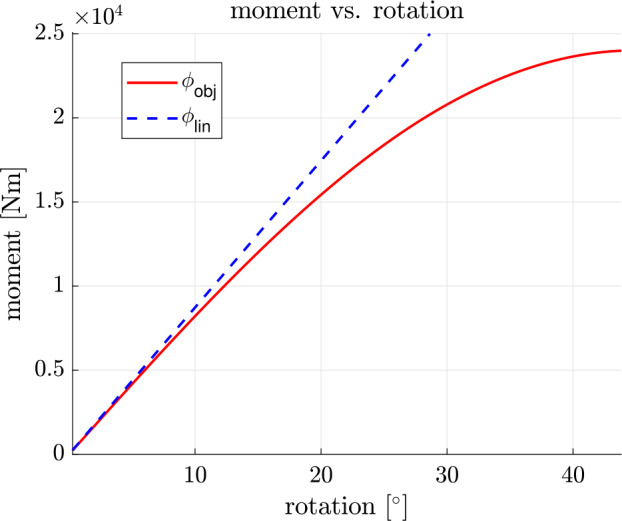
Rotation of the coupling element, depending on the moment. Comparison between a linear deformation measure and the implemented objective, geometrically exact deformation measure

To illustrate the geometrically exact behaviour, we perform a static analysis with a force $\mathbf{F}$ located at the tip of the beam, as shown in Fig. 4. The force is applied in increments of $\Delta \mathbf{F = 0.52} \textbf{ N}$ per load step. The force is increased until a stability onset occurs occurs, i.e. the coupling element stiffness tends to zero and the static solver diverges consequently. Depending on the increasing load, we evaluate the rotation $\phi _{\text{ce}}$ of the node to which the coupling element is attached. The result is plotted in Fig. 5. The dashed blue straight line $\phi _{lin}$ represents the linear relationship between moment and rotation, as would occur with a linear deformation measure, following $\phi _{lin} = \frac{F\cdot l}{c_{66}}$. For the given scenario, it can be seen that the resulting rotation is approximately the same for small angles up to $\phi \approx 5 ^{\circ}$. For larger angles, the geometrically exact deformation measure behaves increasingly softer. This behaviour is plausible and physically more exact than the linear relationship between rotation and load. The representation allows us to perform stability analyses of whole MBS including couplings that experience large deformations. In the linear case, the stability problem is not visible. In an engineering application, this would lead to a more conservative assessment of a component.

#### Verification of the formulation for small deformations

The model illustrated in Fig. 4 is used to verify the coupling element formulation for small displacements and rotations against commercial software. In Sect. 4.2.1, it is shown that, for small rotation angles of the flexible beam, similar behaviour of the geometrically exact and linear deformation measures can be expected.

We compare the maximum tip deflection of the loaded system against ABAQUS/CAE 2019.HF4 for the fixed and flexibly supported cases. The diagonal elasticity matrix of the coupling element ($c_{11}= c_{22}=c_{33}=2 \cdot 10^{6}\frac{\text{N}}{\text{m}}$, $c_{44}=c_{55}=c_{66}=5\cdot 10^{4} \frac{\text{Nm}}{\text{rad}}$) remains unchanged. In ABAQUS, three translational and three rotational spring elements are used with the same elasticity as employed in the coupling element. ABAQUS applies a linear deformation measure in the applied spring elements. A simulation time of $T = 2.0 \text{ s}$ was used with a constant load step of $\Delta \mathbf{F} = 0.15 \text{ N}$. The maximum force applied to the tip equals $\mathbf{F}_{max} = 300 \text{ N}$. Self-weight is again not considered. We determine the results for the fixed case in ABAQUS using a nonlinear Timoshenko beam theory. By showing that the difference of the tip deflection is small (0.0053$\%$) for both clamped beams, we can exclude the beam kinematics as well as the FE discretisation as an influence on the result for the flexibly mounted beam. Table 2 contains the results for both cases. As can be seen here, there are only minor differences for the clamped beam (0.0647%). The flexible support also shows no significant differences (0.2558%) for the small rotations of the coupling and spring elements. Additionally, Table 2 includes the rotation angles of the rotational spring and coupling element. The rotation of the coupling element is $3.4460^{\circ}$. The rotation is thus within the range of small angles shown in Fig. 5, where a similar moment/rotation ratio is to be expected.

**Table 2:** Comparison of the displacement of the loaded tip of the beam, the rotation of the supporting spring/ coupling element and the relative deviation for rigid and flexible soil; determined with DeSiO and ABAQUS

Support	DeSiO	ABAQUS	Deviation [%]
Fixed [mm]	57.110	57.113	0.0053
Rotation angle [°]	-	-	-
Flexible [mm]	658.377	656.697	0.2558
Rotation angle [°]	3.4460	3.4343	0.3407

#### Numerical check of path independence

To numerically demonstrate the path independence of the formulation, the example depicted in Fig. 4 is used again. Of course, this does not correspond to a universally valid proof. A set of three forces ($F_{1}=F_{2}=F_{3}=1\text{ kN}$) is linearly applied in all three spacial directions simultaneously. The forces are then linearly reduced so that the structure should return to its original position.

Table 3 contains the displacements of the beam tip $u_{1}$, $u_{2}$ and $u_{3}$ depending on the loads. After removing the forces, the calculated deflection is zero again. There is also no remaining rotation in the coupling element as this would cause a deflection of the tip, too. This example numerically indicates the path independence by the beam’s return to its initial position and the lack of hysteresis. The analytical proof is outside the scope of this paper.

**Table 3:** Displacement of the beam tip, depending on the load path

F [kN]	$u_{1}$ [m]	$u_{2}$ [m]	$u_{3}$ [m]
[0.0 0.0 0.0]	0.00000000	0.00000000	0.00000000
[0.2 0.2 0.2]	0.43966617	0.43966617	−0.01917738
[0.4 0.4 0.4]	0.88453386	0.88453386	−0.07826113
[0.6 0.6 0.6]	1.33870986	1.33870986	−0.18050220
[0.8 0.8 0.8]	1.80751081	1.80751081	−0.33187553
[1.0 1.0 1.0]	2.29869293	2.29869293	−0.54284693
[0.8 0.8 0.8]	1.80751081	1.80751081	−0.33187553
[0.6 0.6 0.6]	1.33870986	1.33870986	−0.18050220
[0.4 0.4 0.4]	0.88453386	0.88453386	−078261130
[0.2 0.2 0.2]	0.43966617	0.43966617	−0.01917738
[0.0 0.0 0.0]	0.00000000	0.00000000	0.00000000

### Transient and modal analysis of an oscillating structure

**Fig. 6:**
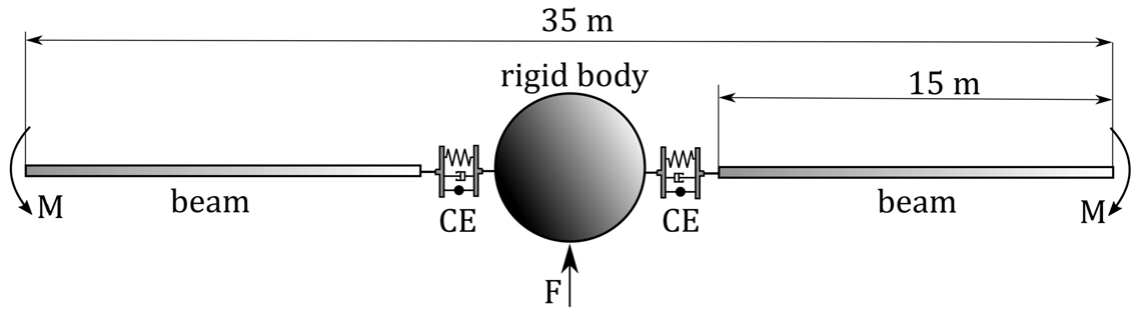
Schematic representation of the MBS resembling a satellite structure

To demonstrate the influence of the coupling element in a transient analysis, we choose a structure that resembles a satellite, as shown in Fig. 6. The structural parameters are chosen freely to model an oscillating system illustrating the dynamic behaviour and the influence of the damping. We chose a dissipation parameter $\alpha = 0.5$ to dissipate energy from the system. Linear and angular momenta are not reduced due to the conservation properties of the damping algorithm. Furthermore, we use a fast Fourier transformation (FFT) to show that the low natural frequencies are not affected by the chosen dissipation parameter.

To model the satellite structure, a rigid body is coupled to two geometrically exact beams. The geometrically exact beams have the properties of a $15.0 \text{ m}$ long and $30.0 \text{ mm} \times 30.0 \text{ mm}$ wide cuboidal titanium body ($\rho _{\text{Ti}} = 4500 \frac{\text{kg}}{\text{m}^{3}}$, $\text{E}_{\text{Ti}} = 120 \text{ GPa}$, $\nu _{\text{Ti}} = 0.3$). We have chosen a discretisation of $n_{el} = 30$ elements per beam. The properties of the rigid body correspond to a solid aluminium sphere with $\rho _{\text{Al}} = 2700 \frac{\text{kg}}{\text{m}^{3}}$ and a radius of $r = 150.0 \text{ mm}$ and thus a mass of $m_{\text{rb}} = 38.88 \text{ kg}$. This represents the bus of the satellite structure. The properties of the coupling element are chosen such that the connection undergoes significant deformation due to the loading of the structure. A diagonal stiffness matrix ($c_{11}=c_{22}=c_{33}=3\cdot 10^{8} \frac{\text{N}}{\text{m}}$, $c_{44}=c_{55}=c_{66}=3\cdot 10^{4} \frac{\text{Nm}}{\text{rad}}$), see Equation (21), is employed. For the mass, we choose the equivalent of an aluminium sphere with a radius $r_{\text{ce}} = 50.0 \text{ mm}$, resulting in a weight of $m_{\text{ce}} = 1.414 \text{ kg}$.

Bending is provoked by applying two moments $M = 500.0 \text{ Nm}$ in opposite directions acting on the tip nodes of the respective beams. These moments are linearly increased over a period of $1.0 \text{ s}$ and then abruptly removed, allowing the system to oscillate freely. The oscillatory motion is superposed onto a transverse rigid-body motion caused by the force $F = 50.0 \text{ N}$ on the rigid body. This is also increased linearly over the period of $1.0 \text{ s}$ and then removed. No gravity has been applied. The total simulation time is $T = 180.0\text{ s}$ with a constant time step of $\Delta t = 10^{-2}\text{ s}$.

**Fig. 7:**
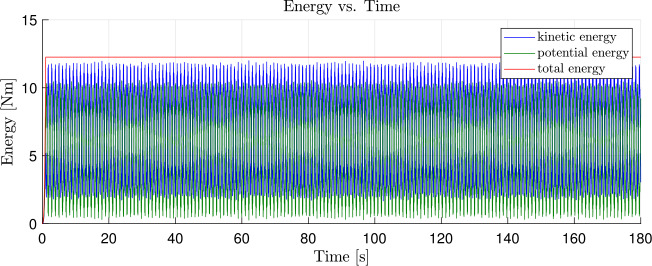
Time history of the energy of the satellite structure with dissipation parameter $\alpha = 0.0$ (Colour figure online)

**Fig. 8:**
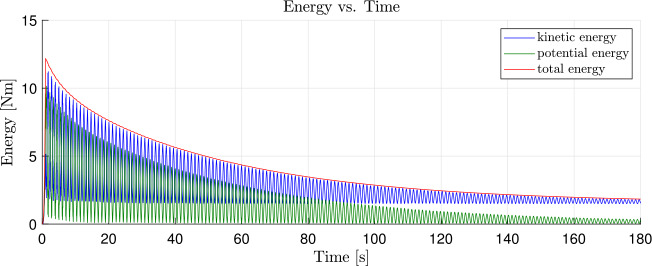
Time history of the energy of the satellite structure with dissipation parameter $\alpha = 0.5$ (Colour figure online)

In a first scenario, see Fig. 7, there is no dissipation. Consequently, there are no damping forces and moments to reduce the internal strain energy of the system. In a second scenario, we applied the strain-rate/stress-rate dependent modified first-order dissipation scheme, as described in Sect. 3.3.1. The dissipation factor $\alpha = \alpha _{\Gamma }= \alpha _{\Omega}$ is set to $\alpha = 0.5$, as used in Equations (55) and (56), such that nonphysical high frequencies are dissipated. Also, the total energy of the system is reduced without affecting the low natural frequencies. The resulting development of potential, kinetic and total energy of the system is shown in Fig. 8. As no gravity is applied in this example, the potential energy consists only of the internal strain energy, stored in the geometrically exact beams and in the coupling elements. The total energy of the system without damping is constant for $t>1.0\text{ s}$ due to the time integration scheme presented, as described in Sect. 2. If damping is employed, the total energy is reduced, as the flapping movement of the satellite structure diminishes. This tends towards a constant value, which is the result of the rigid body movement caused by the force and elastic energy. The remaining elastic energy results from the oscillation of natural frequencies, which do not affect the displacement of the coupling element.

**Fig. 9:**
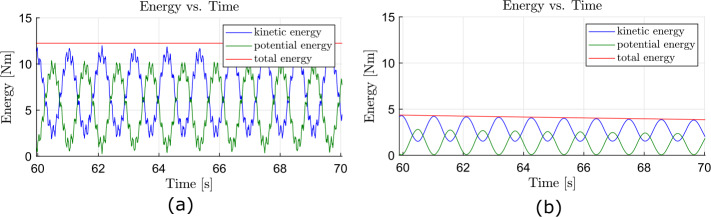
Time history of the energy in the interval between $60.0\text{ s}$ and $70.0\text{ s}$; **(a)** $\alpha = 0.0$, magnified view of Fig. 7; **(b)** $\alpha = 0.5$, magnified view of Fig. 8 (Colour figure online)

The system without damping is subject to vibrations of nonphysical high frequencies. Figure 9 (a) shows a zoomed view into Fig. 7 in the time interval between $60.0\text{ s}$ and $70.0\text{ s}$. A comparison of the same time interval of the damped system, shown in Fig. 9 (b), shows that the high frequency components were dissipated from the oscillation. To prove that the dissipation only reduces the strain energy, we perform another energy analysis after a simulation time of $t = 400.0\text{ s}$. In addition, we introduce a small amount of strain-dependent damping to the geometrically exact beams, with $\chi = \chi _{\Gamma} = \chi _{\Omega} = 0.1$, as governed by Equations (48) and (49). This allows to efficiently dampen the entire stain energy from the system. Consequently, only kinetic energy would remain in the system’s rigid body translation. The total energy after the transient analysis of $400.0\text{ s}$ is determined to be $1.4585 \text{ Nm}$. This corresponds to the analytically calculated total energy $T$ that is present as kinetic energy due to the rigid body movement. This can be determined using a total mass of the system $m_{total} = 214.808\text{ kg}$ and a velocity of $v_{satellite} = 0.1163\frac{\text{m}}{\text{s}}$ to be $T = \frac{1}{2} m_{total}v_{satellite}^{2} = 1.4555 \text{ Nm}$. This corresponds to a deviation of 0.2057%.

**Fig. 10:**
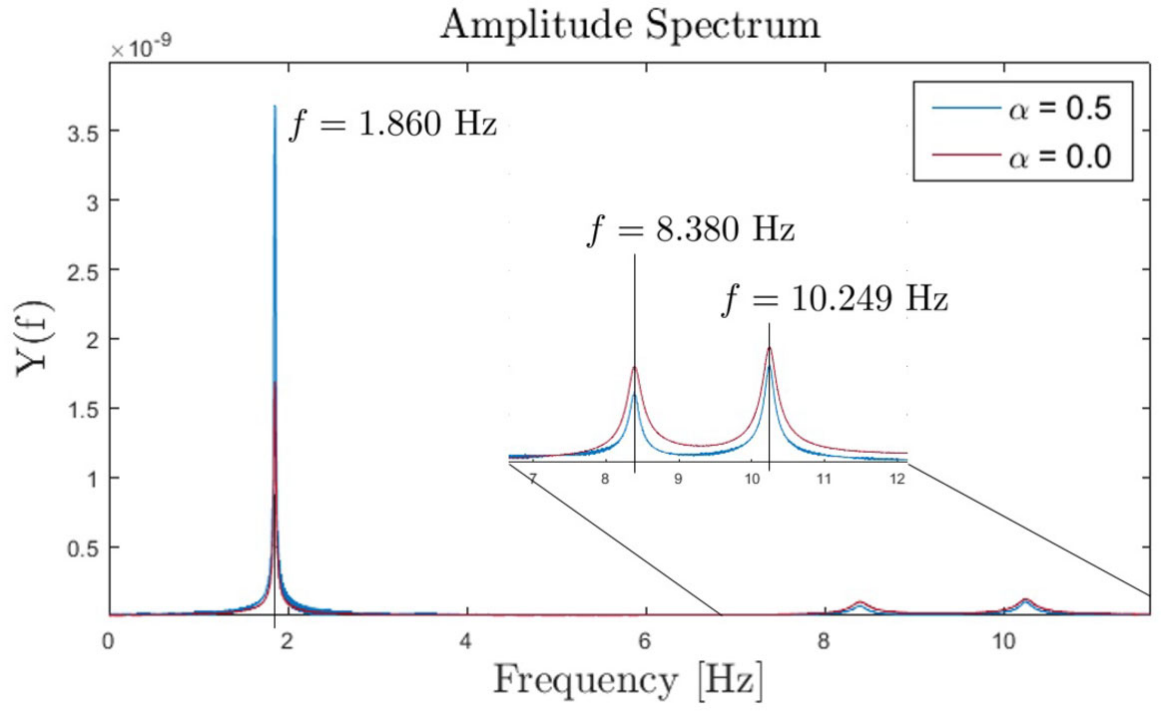
FFT analyses of the satellite beam displacements, blue corresponds to$\alpha = 0.5$, red corresponds to $\alpha = 0.0$ (Colour figure online)

We choose the dissipation parameter $\alpha = 0.5$. To support this choice, we perform an FFT analysis for the system with $\alpha = 0$ and $\alpha = 0.5$ to show that the chosen value has no effect on the low natural frequencies. Still, the total energy of the system is reduced and nonphysical high frequencies are dissipated. Of course, a high dissipation parameter will also affect the lower natural frequencies of a system. To perform the FFT analysis, we use the same transient simulation data as for the energy conservation illustration above, shown in Fig. 8. Figure 10 shows the amplitude spectrum resulting from the FFT analyses. The damped case $\alpha = 0.5$ is plotted in blue and the system without damping $\alpha = 0.0$ in red. The translational displacements of the beam tip were used as input values for the FFT analysis. These oscillate at the lower natural frequencies, which are natural bending modes. As mainly the first natural frequency is excited, the first peak at $f = 1.860 \text{ Hz}$ is more pronounced than the following. It is evident that the chosen dissipation parameter has no influence on the low natural frequencies.

### Modal analysis of a wind turbine considering the soil–structure interaction

We use the coupling element to model the soil–structure interaction of a wind turbine to demonstrate its applicability to a complex mechanical example. Furthermore, the influence of the coupling element on the natural frequencies is evaluated qualitatively. In future research, we will investigate the soil–structure interaction using the coupling element, including mass and damping.

As the soil–structure interaction can affect the structural behaviour of the wind turbine significantly, it is considered in common wind turbine simulation frameworks such as those described in [7–9]. In these simulation packages, the complex mechanical behaviour of the surrounding soil can be idealised by a stiffness matrix coupled to the bottom node of the wind turbine tower. For this purpose, we use the coupling element here.

We choose the example of the *IEA-15-240-RWT* reference wind turbine. The geometry, mass and stiffness data are published in the technical report [32]. The structure is designed to serve as a benchmark and platform for future wind energy developments. In this example, we consider an onshore configuration of the wind turbine, i.e.no substructure is attached below the tower.

**Fig. 11:**
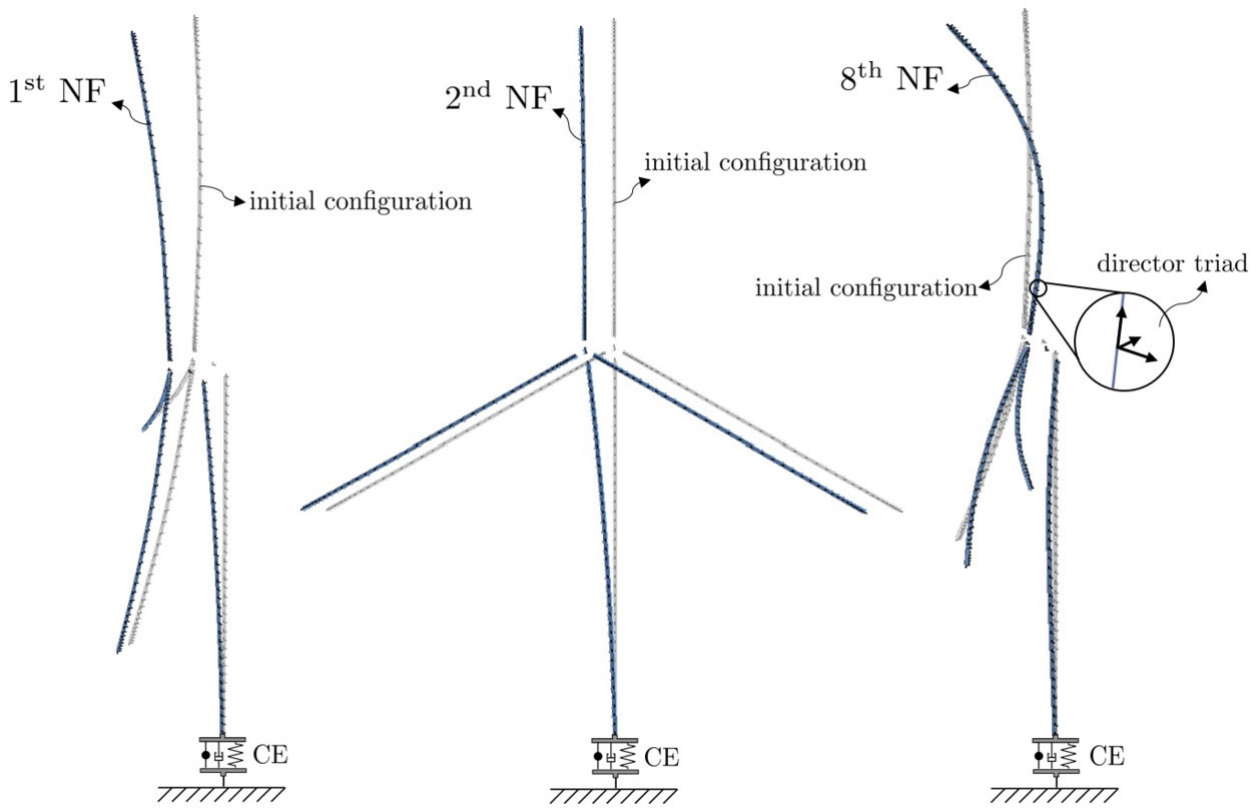
Visualisation of the 1^st^ (left), 2^nd^ (centre) and 8^th^ (right) natural frequencies (NF) considering the flexibly modelled soil

In the MBS simulation, the flexible components of the turbine, the blades and the tower, are modelled using geometrically exact beams. The blades and the tower are each spatially discretised with $n_{el} = 40$ elements. Each element of the respective beam is given the corresponding cross-sectional properties published in the technical report. Since the hub and nacelle are not expected to deform significantly, they are idealised as rigid bodies. In the model considered, the blades are mounted with a pitch angle of $\varphi _{pitch} = 0^{\circ}$. To illustrate the concept, we choose a stiffness matrix with the entries denoted in Table 4. These are realistic values chosen according to the methodology described by Häfele et al. [33]. In Table 5, we compare the first natural frequencies of the wind turbine structure, considering rigid and flexible soils. An illustration of the structure is shown in Fig. 11. Here, the mode shapes corresponding to natural frequencies, in which tower bending is involved, are shown. The director triads in each node are visualised. One node is shown enlarged for clarity. The nondeformed initial configuration of the structure is shown in grey, and the mode shapes are shown in blue. In Table 5 it can be seen that the flexible coupling of the structure leads to a reduction in the natural frequencies. In particular, a large deviation can be seen in the low natural frequencies (1^st^ NF: $7.3398\%$, 2^nd^ NF: $7.5187\%$). The corresponding natural modes are the natural bending modes of the tower (fore-aft, side-to-side), as shown in Fig. 11. Therefore, soil deformation is involved, and the effect on these frequencies is to be expected. The 8^th^ natural frequency also involves a higher tower natural bending mode. Thus, a high deviation regarding the rigid soil can again be seen ($5.6724 \%$).

**Table 4:** Entries in the elasticity matrix of the coupling element used to consider the soil stiffness of a wind turbine foundation

$c_{11}$, $c_{22}[\frac{\text{N}}{\text{m}}]$	$c_{33}[\frac{\text{N}}{\text{m}}]$	$c_{44}$, $c_{55}$, $c_{66}[\frac{\text{Nm}}{\text{rad}}]$	$c_{15}$, $c_{51} [\frac{\text{N}}{\text{rad}}]$	$c_{24}$, $c_{42}[\frac{\text{N}}{\text{rad}}]$
2⋅10^9^	9⋅10^9^	3.0⋅10^11^	−4⋅10^9^	4⋅10^9^

**Table 5:** Comparison of the first natural frequencies (NF) of the *IEA-15-240-RWT* reference wind turbine for rigid and flexible soils

NF [Hz]	1	2	3	4	5	6	7	8
Rigid	0.2161	0.2189	0.5675	0.6447	0.6973	0.9201	0.9338	1.5180
Flexible	0.2003	0.2025	0.5648	0.6403	0.6963	0.9191	0.9326	1.4321
Deviation [%]	7.3398	7.5187	0.4756	0.6878	0.1541	0.1182	0.1237	5.6724

The influence of the flexible soil is a plausible result and will be investigated in more detail in future work, together with the mass of the soil and its damping properties.

## Concluding remarks

The motivation of this work was to develop a general node-to-node coupling element formulation to consider geometrical nonlinearities when connecting components in MBS simulations. The coupling element is a consistent contribution to the mechanical framework presented in Sect. 2. Its properties of objectivity, path independence and the preservation of linear momentum, angular momentum and the total energy are maintained. The coupling considers elasticity, inertia and damping forces.

We described the mechanical derivation, which allows the computation of the elastic forces acting on the respective nodes based on a $6\times 6$ elasticity matrix and considering an objective deformation measure. Inertia forces are considered as an arbitrarily shaped continuum between the coupled nodes. A fully populated inertia matrix can be considered accordingly. Strain/stress dependent damping was implemented following the work of Armero and Romero [28] and Gebhardt et al. [19]. In addition, a modification of this formulation has been shown to result in strain-rate/stress-rate dependent damping, demonstrating that different dissipation functions can be used. This allows the damping to be applied to specific physical problems, e.g.considering damping functions that are material dependent.

Finally, we demonstrated the behaviour of the coupling element. In static analyses, the geometrically exact behaviour was illustrated compared to a linear deformation measure. For small rotation angles, the results were successfully verified against commercial software. In a further example, path independence was indicated numerically. The influence of the damping formulation was demonstrated in transient analyses of an oscillating structure. Finally, the coupling element was employed to model the soil–structure interaction of a wind turbine. A plausible influence on the natural frequencies was shown.

The main limitations of the presented coupling element concerning large deformations of the coupling element. These cannot be modelled physically correctly, in particular due to the linear elastic material properties. As described, large displacements and rotations with moderate deformations can be represented with a nonlinear deformation measure and linear elastic material behaviour. Furthermore, when using the mass properties, it must be taken into account that the mass is not distributed along the length of the element, but remains in the centre of the element when the coupling element is stretched.

In future work, we plan to investigate higher-order dissipation functions. A promising application domain of the coupling element is the more detailed consideration of soil–structure interaction. In particular, the modelling of the soil damping behaviour using the coupling element will be investigated. The elastic blade–hub connection of wind turbines, which is assumed to be rigid in conventional MBS simulations, is also of interest. In this work, we focused on moderate deformations. To also consider large deformations. the application of a nonlinear material model is necessary, which will form further research.
